# MAPK10 Expression as a Prognostic Marker of the Immunosuppressive Tumor Microenvironment in Human Hepatocellular Carcinoma

**DOI:** 10.3389/fonc.2021.687371

**Published:** 2021-08-02

**Authors:** Huahui Li, Yuting Li, Ying Zhang, Binbin Tan, Tuxiong Huang, Jixian Xiong, Xiangyu Tan, Maria A. Ermolaeva, Li Fu

**Affiliations:** ^1^Guangdong Province Key Laboratory of Regional Immunity and Diseases, Department of Pharmacology and Shenzhen University International Cancer Center, Shenzhen University Health Science Center, Shenzhen, China; ^2^Group of Homeostasis and Stress Tolerance, Leibniz Institute on Aging-Fritz Lipmann Institute, Jena, Germany; ^3^Shenzhen University-Friedrich Schiller Universitat Jena Joint PhD Program in Biomedical Sciences, Shenzhen University School of Medicine, Shenzhen, China

**Keywords:** MAPK10, ICAM1, hepatocellular carcinoma, tumor microenvironment, immune cells, immune surveillance, tumor infiltration lymphocytes

## Abstract

Hepatocellular carcinoma (HCC) remains a devastating malignancy worldwide due to lack of effective therapy. The immune-rich contexture of HCC tumor microenvironment (TME) makes this tumor an appealing target for immune-based therapies; however, the immunosuppressive TME is still a major challenge for more efficient immunotherapy in HCC. Using bioinformatics analysis based on the TCGA database, here we found that MAPK10 is frequently down-regulated in HCC tumors and significantly correlates with poor survival of HCC patients. HCC patients with low MAPK10 expression have lower expression scores of tumor infiltration lymphocytes (TILs) and stromal cells in the TME and increased scores of tumor cells than those with high MAPK10 expression. Further transcriptomic analyses revealed that the immune activity in the TME of HCC was markedly reduced in the low-MAPK10 group of HCC patients compared to the high-MAPK10 group. Additionally, we identified 495 differentially expressed immune-associated genes (DIGs), with 482 genes down-regulated and 13 genes up-regulated in parallel with the decrease of MAPK10 expression. GO enrichment and KEGG pathway analyses indicated that the biological functions of these DIGs included cell chemotaxis, leukocyte migration and positive regulation of the response to cytokine–cytokine receptor interaction, T cell receptor activation and MAPK signaling pathway. Protein–protein interaction (PPI) analyses of the 495 DIGs revealed five potential downstream hub genes of MAPK10, including SYK, CBL, VAV1, LCK, and CD3G. Several hub genes such as SYK, LCK, and VAV1 could respond to the immunological costimulatory signaling mediated by the transmembrane protein ICAM1, which was identified as a down-regulated DIG associated with low-MAPK10 expression. Moreover, ectopic overexpression or knock-down of MAPK10 could up-regulate or down-regulate ICAM1 expression via phosphorylation of c-jun at Ser63 in HCC cell lines, respectively. Collectively, our results demonstrated that MAPK10 down-regulation likely contributes to the immunosuppressive TME of HCC, and this gene might serve as a potential immunotherapeutic target and a prognostic factor for HCC patients.

## Introduction

Hepatocellular carcinoma (HCC) is one of the most common fatal malignances of the digestive system and ranks the second leading cause of cancer death in China (1). Moreover HCC remains a devastating malignancy worldwide due to lack of effective therapy for recurrence and metastasis and immune escape (2–4). The formation of immunosuppressive tumor microenvironment and exhaustion of tumor infiltration lymphocytes (TILs) have been implicated in hepatocarcinogenesis. Yet, the molecular mechanisms underlying the evasion of immune surveillance by HCC cells remain largely unclear.

Mitogen-activated protein kinase 10 (MAPK10, also known as JNK3) is a member of the MAP kinase (MAPK) family, and MAPKs play a crucial role in the carcinogenesis and cancer progression by acting as the integration point for multiple biochemical signals (5–7). Moreover, it has been reported that MAPK10 could be activated by the environmental stress-induced dual phosphorylation of its amino acid residues threonine-221 (Thr221) and tyrosine-223 (Tyr223) (8), thus impacting a wide variety of cellular processes, for instance cell proliferation, differentiation, and regulation of gene transcription (9). Over a long period of time in the field of neurobiology, MAPK10 was recognized for its antioxidant properties in ROS scavenging and its protective effects against cerebral ischemic–reperfusion injury (10–12), whereas its molecular functions in oncology and tumor immunology were seldom studied by researchers. Earlier studies from our group have identified organic cation transporter 3 (*OCT3*, also known as *SLC22A3*) as a novel antioxidant gene involved in ROS scavenging in esophageal cancer (13, 14). Importantly, we also revealed that *MAPK10* was the most significantly up-regulated MAPK gene in response to ectopic overexpression of *SLC22A3* (13). Additionally, we have preliminary evidence that MAPK10 was frequently down-regulated in liver cancer patients. In this study, we find that the down-regulation of MAPK10 in HCC patients associates with poor survival prognosis, suggesting that MAPK10 might function as a prognostic factor and a potential therapeutic target for clinical treatment of HCC.

Here, we investigated the expression status of MAPK10 in HCC and used transcriptomics to characterize the impact of MAPK10 expression on the gene expression landscape of the tumor microenvironment in human hepatocellular carcinoma. We used the Cancer Genome Atlas (TCGA) database (https://portal.gdc.cancer.gov/) created by the National Cancer Institute and the National Human Genome Research Institute, to derive data for the bioinformatics analysis of TME gene expression in HCC patients. We utilized the bioinformatics predictions and computer simulations followed by *in vitro* functional assays to characterize the putative role of MAPK10 in HCC tumor microenvironment. We found that high MAPK10 expression associates with increased transcriptional signatures of tumor infiltration lymphocytes (TILs), suggesting that MAPK10 could be implicated in the recruitment of TILs into the TME of HCC. We also performed *in vitro* validation tests to propose that MAPK10 regulates TMEs’ immune status *via* expression of ICAM1. Our findings provide novel testable hypotheses for designing better prognostic strategies and potential immunotherapy strategies for HCC in clinical practice.

## Methods and Materials

### Transcriptome Analysis of the HCC Data Obtained From the TCGA Database

Transcriptome sequencing data of a total of 374 HCC patient samples and their corresponding adjacent normal tissues were downloaded from The Cancer Genome Atlas (TCGA) database (https://portal.gdc.cancer.gov/). For the estimation of gene expression by using RNA-seq data, the TCGA-liver hepatocellular carcinoma (TCGA-LIHC) project used the fragments per kilobase of exon model per million reads mapped (FPKM) normalization method. The mean expressional level of MAPK10 mRNA in non-tumor liver tissue of HCC patients was 0.115694 (FPKM), which was assigned in this study as the cutoff point for dividing HCC patients into two groups: the HCC patients with MAPK10 expression greater than 0.115694 (FPKM) were considered as high-MAPK10 expression group. Conversely, the patients with MAPK10 expression below 0.115694 (FPKM) were defined as low-MAPK10 expression group. Pan-cancer analysis using TCGA data was conducted in a web server of Tumor IMmune Estimation Resource 2.0 (TIMER 2.0, http://timer.comp-genomics.org/). The survival analysis of the patients belonging to the TCGA-LIHC project was conducted by use of an open source online Kaplan–Meier plotter (https://kmplot.com/analysis/). Data analysis, statistics analysis, and data visualization were executed by R programming language.

### Bioinformatics Analysis Based on the Data From the TCGA Database

Initially, R package of ESTIMATE (Estimation of STromal and Immune cells in MAlignant Tumor tissues using Expression data) (15) was utilized to calculate the scores for tumor cells, immune cells, and stromal cells in the TME of the HCC patients included in the TCGA database. We obtained the following three values from ESTIMATE software: stromal scores, immune scores, and ESTIMATE scores. These three values have the following meaning: the higher the stromal scores are, the higher the content of stromal cells is in the tumor microenvironment. The same relationship is true for the immune scores and the content of immune cells in the TME. Immune score of each patient plus the corresponding stromal score derives an ESTIMATE score for each individual patient. The following equation was utilized to calculate the ESTIMATE score for each patient:

ESTIMATE score of each patient=Immune score of each patient + the corresponding stromal score.

The higher the ESTIMATE scores are, the higher are the contents of stromal cells and immune cells in a given tumor microenvironment and the lower is the content of tumor cells. The tumor score was calculated by subtracting the individual ESTIMATE score of each patient from the maximal ESTIMATE value of the entire cohort by use of the following equation:

Tumor score=maximal value of ESTIMATE score in HCC cohort- ESTIMATE score of each liver cancer patient.

Therefore, tumor scores represented the content of tumor cells in the tumor microenvironment of each patient. Furthermore, CIBERSORTx software (https://cibersortx.stanford.edu/) was utilized to evaluate the composition of specific immune cells in the tumor microenvironment (16, 17).

Moreover, the R package of GSVA (Gene Set Variation Analysis) (18) using gene-centric single sample Gene Set Enrichment Analysis (ssGSEA) method (19) was utilized to analyze the immune activity of the tumor microenvironment for each cancer patient with HCC. The 29 immune-associated gene sets used in the ssGSEA analysis and representing a variety of immune cell types and functions were obtained from a previous scientific report (20). The immune activity analysis workflow is presented in the **Supplementary Figure 1A**. Using transcriptome sequencing data of HCC patients as an input and ssGSEA method in GSVA package, we obtained the immune landscape and immune profiles of HCC patients, which cover five major immune-associated aspects including content of distinct immune cells, expression of major histocompatibility complex class I/II (MHC-I/II), immunoreactive intensity of IFN response, inflammatory activity and cytolytic activity. Subsequently, according to immune landscape and immune profiles of HCC patients, we classified these patients into three categories (**Supplementary Figures 1B, C**) using the hierarchical clustering approach in the R programming environment. These three categories were high immune activity, medium immune activity, and low immune activity respectively. The group of HCC patients (Cluster 1 in **Supplementary Figure 1C**, n = 185) who had low content of distinct immune cells, low expression of major histocompatibility complex class I/II (MHC-I/II), low immunoreactive intensity of IFN response, inflammation and cytolytic activity were assigned as HCC patients with low immune activity. Another group of HCC patients (Cluster 3 in **Supplementary Figure 1C**, n = 81) who had high content of distinct immune cells, high expression of major histocompatibility complex class I/II (MHC-I/II), high immunoreactive intensity of IFN response, inflammation and cytolytic activity were defined as HCC patients with high immune activity. The third group of HCC patients (Cluster 2 in **Supplementary Figure 4C**, n = 108) who had medium content of distinct immune cells, medium expression of major histocompatibility complex class I/II (MHC-I/II), medium immunoreactive intensity of IFN response, inflammation and cytolytic activity were classified as HCC patients with medium immune activity.

Additionally, a gene list of 2,498 human immunity-related genes was downloaded from the immunology database and analysis portal (ImmPort) (https://www.immport.org/shared/genelists). We next used custom R scripts to measure the fold change of these genes in MAPK10-high and -low expressing patients by dividing the average expression level of each immunity-related gene in the low-MAPK10 group by its corresponding average expression level in the high-MAPK10 group, the below equation was utilized:

$$\text{Average}\;\text{fold}\;\text{change}=\frac{\text{an}\;\text{average}\;\text{expression}\;\text{level}\;\text{of}\;\text{each}\;\text{immunity}-\text{related}\;\text{gene}\;\text{in}\;\text{low}-\text{MAPK}10\;\text{group}}{\text{its}\;\text{corresponding}\;\text{average}\;\text{expression}\;\text{level}\;\text{in}\;\text{high}-\text{MAPK}10\;\text{group}}.$$

To outline the differentially expressed immune-associated genes (DIGs), we selected the immunity-related genes with average log2(fold change) of at least 0.585 and Q value (also known as adjusted *P* value) of less than 0.05.

To achieve the optimal data visualization of the DIG expression in a heat map format, it is currently common to transform the matrix of original gene expression onto linearly distributed data in a form of log2 scale or log10 scale. However, the visualization of the linearly distributed data may be distorted by the presence of the outliers. To mitigate this potential distortion, we first transformed the original gene expression levels into log2 scale by using built-in mathematic function of “log2()” in the R programming environment and next transformed these values into standardized normal distribution by using built-in mathematic function of “scale()” in R script and used the resulting data for the graphical plotting. Besides, gene oncology (GO) enrichment and Kyoto Encyclopedia of Genes and Genomes (KEGG) pathway enrichment analysis were performed to define the biological functions of these differentially expressed immune-associated genes. Prediction of protein–protein interaction (PPI) was carried out in STRING (Search Tool for the Retrieval of Interacting Genes/Proteins, https://string-db.org/) database. Furthermore, we also used a plug-in called Cytohubba in Cytoscape software to predict additional strong interactors among the differentially expressed immune-associated genes, which might represent the potential downstream targets of MAPK10.

### Cell Culture

Human hepatocellular carcinoma cell lines HepG2 and Huh-7 were obtained from the American Type Culture Collection (ATCC). HepG2 cells was grown in Dulbecco’s Modified Eagle’s medium (DMEM) containing 10% fetal bovine serum (FBS), and Huh7 cells were cultured in RPMI-1640 medium (Gibco, Thermo Fisher Scientific, lnc., Waltham, MA, USA) supplemented with 10% fetal bovine serum (Gibco, Thermo Fisher Scientific, lnc.) and penicillin (100 U/ml)–streptomycin (0.1 mg/ml). Additionally, culture medium for lentivirus stably infected MAPK10-overexpressing HepG2 cells and MAPK10-deficient Huh7 cells contained 1.0 μg/ml puromycin (Sigma-Aldrich, Germany). All the cells received routine culture in an incubator with 5% carbon dioxide at 37°C.

### Construction of Lentivirus Stably Infected MAPK10-Overexpressing and MAPK10-Deficient HCC Cells

One day before transfection, 3–7 × 10^6^ HEK293T lentiviral packaging cells were seeded onto a 10 cm^2^ petri dish in culture medium supplemented with 10% fetal bovine serum. Subsequently, the cells were incubated overnight at 37°C, 5% CO_2._ At the time of transfection, cell density should be 95–99% confluent. We brought Opti-MEM I Reduced Serum Medium (Gibco Corporation) to room temperature and prepared Tube A and Tube B as described in the following recipe. Tube A consisted of 0.5 ml Opti-MEM I Reduced Serum Medium and 20 μl Lipofectamine 3000 Transfection Reagent (Invitrogen Corporation, Cat. No: L3000015), while Tube B consisted of 0.5 ml Opti-MEM I Reduced Serum Medium, 20 μl P3000 Enhancer Reagent (Invitrogen Corporation, Cat. No: L3000015), 2.5 μg envelope expressing plasmid pMD2.G, 2.5 μg packaging plasmid psPAX2, and 5.0 μg lentiviral transfer plasmid carrying specific sequence such as pLv-MAPK10 (cloned in our lab), pLv-puro (Inovogen Corporation, Cat. No: VL3001), pLKO.1-puro (Sigma-Aldrich, Cat. No: SHC001), and pLKO.1-shMAPK10 (Sigma-Aldrich, Cat. No: TRCN0000196303). Additionally, the protein coding region of MAPK10 variant 1 (NM_001318069.1) was PCR amplified from MIHA cells and cloned into the pLV-puro vector by NotI and EcoRI enzyme digestion and ligation. The target sequence of the shRNA-MAPK10 in the pLKO.1-shMAPK10 plasmid in this study was 5′-CCGGGAATTAGACCATGAGCGAATGCTCGAGCATTCGCTCATGGTCTAATTCTTTTTTG-3′. Moreover, we could slightly adjust the amount of each component according to the area occupied by transfected cells. To prepare lipid–DNA complexes, we transferred the contents of Tube A to Tube B and mixed gently. Subsequently, we incubated the complexes for 20 min at room temperature. Prior to adding complexes to the cells, we removed 50% medium (about 6 ml), leaving a total of 6 ml. We added 3 ml of lipid–DNA complex to the plate, taking care to dispense liquid against the plate wall to avoid disrupting cells and gently swirl the plate to distribute the complex. Subsequently, HEK293T cells were incubated for 16 h in the incubator at 37°C and 5% CO_2_. At 16 h post transfection, we refreshed culture medium. The overnight culture medium containing lipid–DNA complexes was carefully removed and replaced with fresh culture medium supplemented with 30% heat-inactivated fetal bovine serum and penicillin (100 U/ml)–streptomycin (0.1 mg/ml). It was a selectable option to add 1/500 volume of the TiterBoost reagent (GeneCopoeia Corporation) to the culture medium and continue incubation in the CO_2_ incubator at 37°C. Plasmid-transfected HEK293T cells were returned to the incubator and cultured for 24 h at 37°C, 5% CO_2_. At the same time, target HCC cells such as HepG2 and Huh7 were inoculated into 6 cm^2^ petri dish. Approximately 24 or 48 h post transfection, we collected 12 ml of lentivirus containing supernatant in sterile capped tubes. Then we filtered the crude lentiviral supernatant using a 0.45 μm polyethersulfone (PES) filters (low protein-binding filters) to remove cellular debris and to improve the purification and quality of crude lentivirus. The purified supernatant was transferred to a sterile container and one volume of Lenti-X Concentrator (TAKARA Corporation, Cat. No: 631231) was combined with three volumes of the purified supernatant. The mixture was homogenized by gentle inversion and incubated at 4°C overnight. We centrifuged collected lentiviral samples at 1,500 × g for 50 min at 4°C and removed the supernatant carefully to avoid disturbing the precipitated pellets at the bottom of the tubes. The titer of harvested lentiviral stocks was determined conveniently by qRT-PCR using Lenti-Pac HIV qRT-PCR Titration Kits (GeneCopoeia Corporation. Cat. No: HPR-LTK-050). The concentrated and purified lentiviral particles with the magnitude of over 1.0 × 10^8^ titration were gently resuspended in 4 ml complete culture medium containing 5.0 μg/ml polybrene by pipetting up and down. The pellets were subsequently used to infect the target cells in a 10 cm^2^ petri dish. Approximately 72 h post-infection, fresh culture medium supplemented with 30% fetal bovine serum and 1.0 μg/ml puromycin was used to culture the lentivirus-infected target HCC cells. Only cells contacting viral particles were able to survive and propagate under the puromycin selection. In this experiment, all cell culture vessels and pipette tips were treated with 10% bleaching solution. After several generations of culturing, the puromycin-resistant HCC cells were considered to be infected by lentivirus successfully. We verified the gene expressional level of MAPK10 in those HCC cell lines *via* PCR and western blotting assay.

### Quantitative Real-Time PCR

Total RNA was extracted from lentivirus stably infected HCC cell lines with MAPK10 overexpression or deficiency using Universal RNA Extraction Kit (Takara Biomedical technology Co., Ltd., Beijing, China) according to the manufacturer’s instruction. All traces of genomic DNA fragments were removed from the total RNA samples using deoxyribonuclease I (Sigma-Aldrich, Germany). Subsequently, the cDNA was synthesized by reverse transcription-polymerase chain reaction (RT-PCR) assay using PrimeScript™ RT Master Mix (Takara Biomedical technology Corporation). The level of MAPK10 expression in HCC cell lines was determined by quantitative real-time polymerase chain reaction (qPCR) using iTaq™ universal SYBR^®^ Green supermix (Bio-Rad, USA) and CFX96 Touch™ real-time system (Bio-Rad, USA). An endogenous internal control designed based on human 18S rRNA was amplified for each sample. Relative expressional level of MAPK10 and ICAM1 in HCC cell lines was determined by 2^(-ΔΔCt)^ method after the cycle number at threshold (CT value) of each target gene was normalized by subtracting the CT value of the corresponding endogenous internal control. The following primer sequences were used: 18S rRNA sense primer, 5′-CTCTTAGCTGAGTGTCCCGC-3′ and antisense primer 5′-CTGATCGTCTTCGAACCTCC-3′. The product size of 18S rRNA is 249bp. ICAM1 sense primer, 5′-ATGCCCAGACATCTGTGTCC-3′ and antisense primer 5′-GGGGTCTCTATGCCCAACAA-3′. The product size of ICAM1 is 112 bp. Endogenous MAPK10 sense primer, 5′-CTTCCCAGATTCCCTCTTCC-3′ and antisense primer 5′-GCTGGGTCATACCAGACGTT-3′. The product size of endogenous MAPK10 in Huh7-pLKO and Huh7-shMAPK10 is 162 bp. Exogenous MAPK10 sense primer, 5′-GGACGTGTACCTGGTCATGG-3′ and antisense primer 5′-ATGCCGCACAGCATCTGATA-3′. The expected product size of exogenous MAPK10 in HepG2-puro and HepG2-MAPK10 cells is 111 bp. The cDNA synthesized in reverse transcription PCR was diluted with two volumes of nuclease-free water. We prepared the following PCR reaction mixture in a specific optical tube, 10 µl of SYBR Green Mix (2×), 1.0 µl of diluted cDNA, 1.0 µl of forward primer (10.0 µmol/L), 1.0 µl of reverse primer (10.0 µmol/L), and 7.0 µl of nuclease-free water. The qPCR procedure consisted of initiation at a temperature of 95°C for 30 s prior to a series of 40 thermal cycles including denaturation at a temperature of 95°C for 15 s, annealing at a temperature of 57°C for 30 s and elongation at a temperature of 72°C for 30 s. The melting curve analysis was conducted in all cases.

### Western Blot

Western blot analyses in this study were executed according to the standard protocol. Total protein extract was loaded onto 12% SDS-PAGE gel. After electrophoresis in 12% SDS-PAGE gels, proteins were transferred to nitrocellulose membranes. Blots were blocked for 2 h in 0.5% TBST buffer containing 5% BSA. Subsequently, the membranes were incubated with primary antibodies in the shaker at 4°C overnight. The following primary antibodies were used: Recombinant rabbit monoclonal MAPK10/JNK3 antibody (1:1,000) (Abcam Corporation, Cat. No: ab126591), mouse actin antibody (1:1,000) (Cell signaling Technology, USA, Cat. No: #3700). Detailed information of western blotting could be found in the previous papers we published (21–23).

### Statistical Analysis

Data analysis, data visualization (graphical plotting), and statistical analysis were conducted in open-source R studio software using object-oriented R language in the Windows operating system. Statistical analysis of Chi-square test for the association of MAPK10 expression and the immune activity was performed using Statistical Package for the Social Sciences (SPSS, version 24.0). Survival analysis to assess the prognostic effects of MAPK10 expression in HCC patients was conducted by using Kaplan–Meier plots and log-rank tests. Statistical comparison of any two non-normal distribution groups was executed by using Wilcoxon test, while Kruskal test was implemented for the statistical analysis of three or more non-normal distribution groups. The data were presented as mean ± standard deviation (SD) of triplicated independent experiment. *P* values below 0.05 were considered statistically significant.

## Results

### MAPK10 Is a Potential Prognostic Factor for the Survival of Cancer Patients With HCC

**Figure 1:**
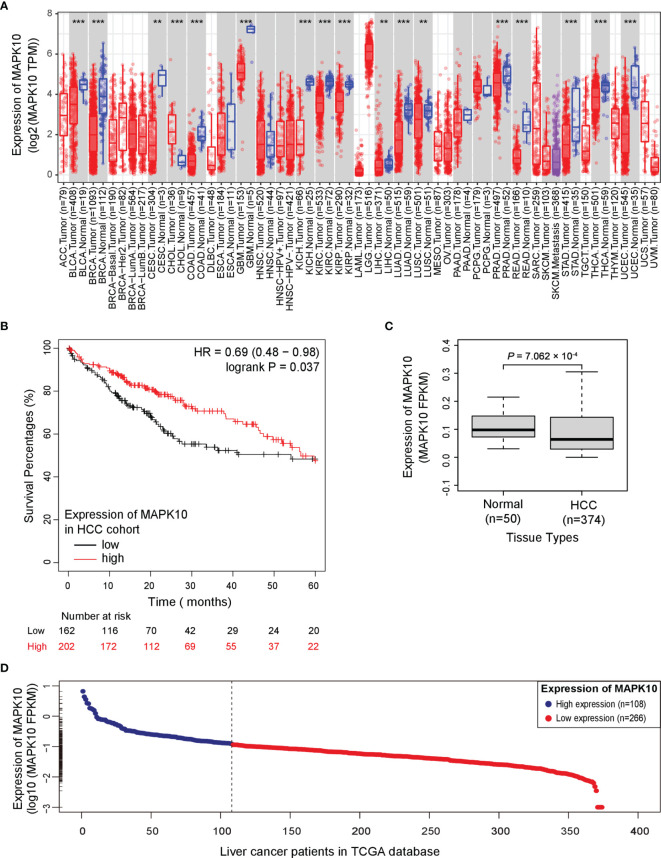
Potential tumor suppressor gene (TSG) *MAPK10* is a prognostic factor for the survival of cancer patients with HCC. **(A)** The pan-cancer analysis using TCGA data was conducted by a web server of Tumor IMmune Estimation Resource 2.0 (TIMER 2.0, http://timer.comp-genomics.org/) showing down-regulation of MAPK10 in a variety of solid tumors. Detailed information describing pan-cancer analysis can be obtained from the following link (http://timer.comp-genomics.org/). **(B)** The survival analysis in a Kaplan–Meier plotter web interface (https://kmplot.com/analysis/) using data from the TCGA database was utilized to compare the five-year survival of HCC patients with low expression of MAPK10 to that of the patients with high MAPK10 expression (HR(hazard ratio) = 0.69, log-rank *P* = 0.037). **(C)** The mRNA level of MAPK10 was compared between human HCC tissues and non-cancerous (normal) liver tissues by use of the Wilcoxon test method (Wilcoxon Test, *P* = 7.062 × 10^−4^). **(D)** After the expression value of MAPK10 in HCC patients was transformed onto Log10 scale by use of a built-in mathematic function of “log10()” in the R programming environment, the mRNA level of MAPK10 was found to decrease in a linear manner. Because the mean value of 0.115694 (FPKM) in non-tumor tissue was assigned as the cutoff for dividing HCC patients into two groups, those HCC patients with MAPK10 expression greater than the cutoff value were defined as high-MAPK10 group (shown as blue dots on the left-hand side, n = 108) whereas the patients with MAPK10 expression less than the cutoff value were defined as low-MAPK10 group (shown as red dots on the right-hand side, n = 266). **P<0.01; ***P<0.001.

To analyze the prognostic significance of MAPK10 expression, pan-cancer analysis using TCGA data was conducted utilizing the web interface of the Tumor IMmune Estimation Resource 2.0 (TIMER 2.0, http://timer.comp-genomics.org/).The pan-cancer analysis showed that MAPK10 was extensively down-regulated in a wide variety of solid tumor tissue (**Figure 1A**). The detailed information describing the pan-cancer analysis approach can be obtained at the following URL (http://timer.comp-genomics.org/). We next performed the Kaplan–Meier survival analysis combined with log-rank tests using the HCC patient data available from the TCGA database. For this analysis, the patients were classified into high-MAPK10 and low-MAPK10 expression groups by using the Maximally Selected Rank Statistics (MSRS) algorithm. We found that the five-year overall survival rate in HCC patients with low expression of MAPK10 was significantly reduced compared to patients with high expression of MAPK10 [HR (hazard ratio) = 0.64, log-rank *P*= 0.037; **Figure 1B**)]. The expression values of MAPK10 in a form of FPKM in both liver cancer tissues and adjacent normal tissues were transformed onto Log10 scale. We found that the mean value of MAPK10 mRNA expression in surrounding non-tumor tissue of HCC was 0.115694 (FPKM). Moreover, we revealed that the mRNA levels of MAPK10 in HCC tumor tissues were significantly reduced compared to non-tumor tissues (Wilcoxon test, *P* = 7.062 × 10^-4^; **Figure 1C**). This result suggested that down-regulation of MAPK10 was a common feature of patients with HCC. Furthermore, this mean value of 0.115694 (FPKM) in non-tumor tissue was assigned as the cutoff for dividing the HCC patients into two groups: the patients with MAPK10 expression greater than the cutoff value were defined as the high-MAPK10 group (shown as blue dots on the left-hand side in **Figure 1D**, n = 108). *Vice versa*, the patients with MAPK10 expression less than the cutoff value were defined as the low-MAPK10 group (shown as red dots on the right-hand side in **Figure 1D**, n = 266). Meanwhile, after the expression values of MAPK10 in HCC patients were transformed onto log10 scale, the mRNA level of MAPK10 in liver cancer tissues decreased in a linear manner (**Figure 1D**). Even though physiological and pathophysiological processes in human body are usually regulated by non-linear dynamic mechanisms, the transformation from non-linear to linear representation has frequently been utilized to characterize the complexity of signal transduction and the changes of gene expression. Therefore in the subsequent changes analyses in this study, we would perform linear transformation using log10 scale or log2 scale if necessary, especially when the changes between MAPK10 expression and genes of interest is analyzed. The above results suggested that MAPK10 expression is an important prognostic factor of the survival of HCC patients.

### High *MAPK10* Expression Associates With Increased Transcriptomic Scores of Stromal and Immune Cells But Decreased Scores of Cancer Cells in the TME of HCC Patients

**Figure 2:**
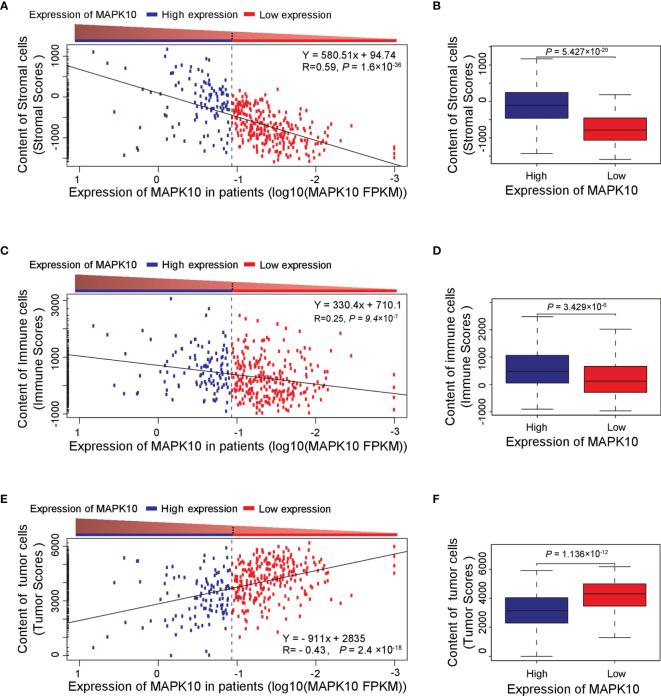
High *MAPK10* expression correlates with the increased content of stromal and immune cells and decreased proportion of cancer cells in the TME of HCC. The Pearson correlation method was used to correlate MAPK10 expression in HCC patients with the abundance of stromal cells **(A)** (Correlation coefficient R^2^ = 0.59. P = 1.6 × 10^−36^), immune cells **(C)** (Correlation coefficient R^2^ = 0.25. P = 9.4 × 10^−7^) and tumor cells **(E)** (Correlation coefficient R^2^ = -0.43. P = 2.4×10^−18^) in the TME. Additionally, the relationship between MAPK10 expression and these parameters was demonstrated through the analysis by Wilcoxon Test for stromal cells, **(B)** (Wilcoxon Test, *P* = 5.427×10^−20^), for immune cells **(D)** (Wilcoxon Test, *P* = 3.429×10^−6^) and for tumor cells. **(F)** (Wilcoxon Test, *P* = 1.136 × 10^−12^) after the patients were divided into high-MAPK10 group (n = 108) and low-MAPK10 groups (n = 266) as described in the *Materials and Methods* section.

Because our previous assessment revealed that MAPK10 might be an important prognostic factor for the survival of patients with HCC, we next explored whether MAPK10 had lasting implications on the TME of HCC. ESTIMATE software was utilized to evaluate the transcriptomic scores reflective of the abundance of tumor cells, immune cells, and stromal cells in the TME. We uncovered that the decrease of MAPK10 expression in HCC patients was positively correlated with the reduction of stromal cell gene expression signatures (correlation coefficient R = 0.59. P = 1.6 × 10^−36^; **Figure 2A**), suggesting that the expression of MAPK10 in the development and progression of HCC might be associated with the differential abundance of cancer-associated fibroblasts (CAFs) or other stromal cells (24–26). The significant reduction of stromal cell signatures in the low-MAPK10 group could be confirmed by separating the HCC patients into the MAPK10 high and MAPK10 low groups (as explained previously) and performing cumulative scoring (Wilcoxon test, *P* = 5.427 × 10^−20^; **Figure 2B**). Furthermore, the decline of MAPK10 expression in HCC patients was positively correlated with the decreased scores of immune cells, suggesting that MAPK10 could facilitate the infiltration of immune cells into the TME (correlation coefficient R = 0.25. P = 9.4 × 10^−7^; **Figure 2C**). Also cumulative scoring showed that HCC patients in the high-MAPK10 group had significantly higher immune cell expression signatures than those in the low-MAPK10 group (Wilcoxon test, *P* = 3.429 × 10^−6^; **Figure 2D**). Intriguingly, the expression of MAPK10 in HCC patients was negatively correlated with the scores of tumor cells, illustrating that MAPK10 might exert anti-tumor effects in HCC (correlation coefficient R = −0.43. *P* = 2.4 × 10^−18^; **Figure 2E**). HCC patients with high expression of MAPK10 had gene expression profiles consistent with lower numbers of cancer cells than those with low MAPK10 expression also per cumulative scoring (Wilcoxon test, *P* = 1.136 × 10^−12^; **Figure 2F**). Collectively, our results showed that differential MAPK10 expression associates with gene expression profiles that are indicative of the distinct cell composition of the liver cancer microenvironment.

### *MAPK10* Expression Correlates With Immune Activity Signatures in the TME of HCC Patients

**Figure 3:**
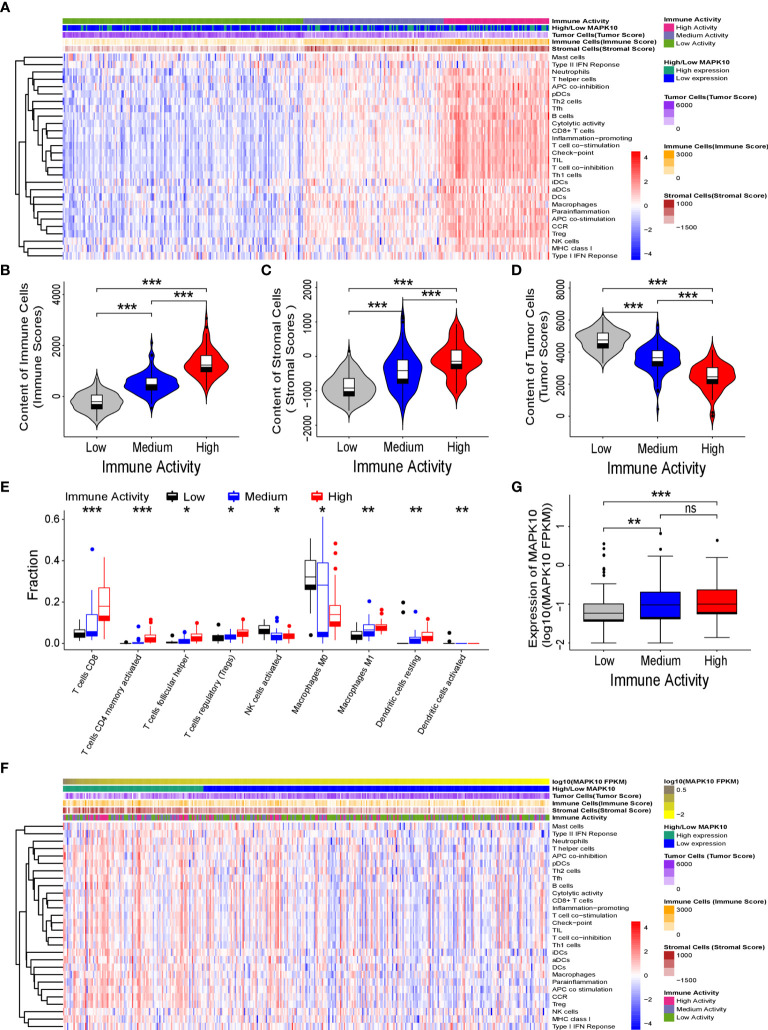
*MAPK10* expression correlates with immune activity of the tumor microenvironment in HCC patients. As described in the *Materials and Methods* section, the immune landscape and immune profiles of HCC patients including immunoreactive intensity of IFN response, inflammation and cytolytic activity were obtained by using ssGSEA (single sample gene set enrichment analysis) in the GSVA (Gene Set Variation Analysis) R package. On the basis of the immune landscape and immune profiles and by using hierarchical clustering, HCC patients were divided into three groups that were corresponding to low, medium, and high immune activity. Then the heat map plotting was used to visualize immune activity of HCC patients and the abundance of immune cells, stromal cells and tumor cells in the liver cancer microenvironment **(A)**. The relationship between immune activity and these parameters was demonstrated by statistical analysis by Wilcoxon Test for immune cells **(B)** (Wilcoxon Test, *P* < 0.001), for stromal cells **(C)** (Wilcoxon Test, *P* < 0.001), and for tumor cells **(D)** (Wilcoxon Test, *P* < 0.001). The predicted cellular compositions of specific immune cells in the HCC microenvironment were obtained by using transcriptome sequencing data of the TCGA database as an input into the CIBERSORTx software (https://cibersortx.stanford.edu/). The statistical analysis by Wilcoxon test uncovered that immune landscape of the TME in the HCC cohort demonstrated significant enrichment of expression signatures representative of CD8^+^ T cells, activated memory CD4^+^ T cells, resting dendritic cells, NK cells and the conversion of macrophages from M0 type to M1 type **(E)** (Wilcoxon test, *P* < 0.05). In order to analyze the link between MAPK10 expression and immune activity, HCC patients were ranked by MAPK10 expression in a descending order **(F)**, and Wilcoxon test was used to correlate immune activity of HCC patients with the expression of MAPK10 **(G)** (Wilcoxon Test, *P* < 0.01). *P<0.05; **P<0.01; ***P<0.001; ns, not significant.

Because our previous results indicated that MAPK10 might facilitate the infiltration of immune cells into the TME, we intended to analyze the immune activity of the TME in patients with HCC by transcriptomics. To confirm whether the immune activity signatures would vary among TMEs of individual HCC patients, GSVA (Gene Set Variation Analysis) R package using ssGSEA (single sample gene set enrichment analysis) method was employed to access the immune landscape and immune profiles through immune gene expression signature of each HCC patient. A schematic diagram to analyze the correlation between MAPK10 expression and immune activity is shown in **Supplementary Figure 1A**. Subsequently, we divided HCC patients into three groups according to their immune gene expression landscape and immune gene expression profiles using hierarchical clustering method in R programming environment (**Supplementary Figure 1B**). The group of HCC patients (Cluster 1 in **Supplementary Figure 1C**, n = 185) who had low expression scores of distinct immune cells, low expression of major histocompatibility complex class I/II (MHC-I/II), low expression of the genes involved in the IFN response, inflammation and cytolytic activity were assigned as HCC patients with low immune activity. Another group of HCC patients (Cluster 3 in **Supplementary Figure 1C**, n = 81) who had high transcriptional scores of distinct immune cells, high expression of major histocompatibility complex class I/II (MHC-I/II), high expression of the genes involved in the IFN response, inflammation and cytolytic activity were defined as HCC patients with high immune activity. The third group of HCC patients (Cluster 2 in **Supplementary Figure 1C**, n = 108) who had medium content of distinct immune cells, medium expression of major histocompatibility complex class I/II (MHC-I/II), medium expression of the genes involved in the IFN response, inflammation and cytolytic activity were classified as HCC patients with medium immune activity. The detailed workflow of the classification process is described in **Supplementary Figure 1** and in *Materials and Methods*. We observed that when HCC patients were grouped by the transcriptomically defined immune activity of the liver cancer microenvironment, the lower immune activity correlated with lower MAPK10 expression, reduced scores of tumor infiltration lymphocytes (TILs) and stromal cells, but increased scores of cancer cells (**Figure 3A**). Interestingly, many specific immune pathways detected in the TME and correlated with MAPK10 expression belong to lymphocyte function (**Figure 3A**), suggesting that immune cells that potentially infiltrate the TME of HCC in a MAPK10 dependent manner might be lymphocytes (TILs). Indeed, the statistical analysis showed that the expression of MAPK10 was significantly correlated with the expression of immune genes in the liver cancer microenvironment (Chi-square *P* < 0.001; **Table 1**). Moreover, HCC patients with high immune activity had significantly higher scores of immune cells than patients with medium immune activity (Wilcoxon test, *P* < 0.001; **Figure 3B**), while HCC patients with medium immune activity had significantly higher immune cell scores than those with low immune activity (Wilcoxon test, *P* < 0.001; **Figure 3B**). Meanwhile, HCC patients with high immune activity had significantly higher scores of stromal cells compared to patients with medium immune activity (Wilcoxon test, *P* < 0.001; **Figure 3C**), and HCC patients with medium immune activity had significantly more stromal cells than those with low immune activity (Wilcoxon Test, *P* < 0.001; **Figure 3C**). Finally, HCC patients with high immune activity had significantly reduced scores of cancer cells than those with medium immune activity (Wilcoxon test, *P* < 0.001; **Figure 3D**), and comparably significant difference was observed between HCC patients with medium immune and low immune activity (Wilcoxon test, *P* < 0.001; **Figure 3D**). Collectively, HCC patients with higher immune activity (assigned *via* transcriptomic analysis) presented gene expression signatures consistent with higher numbers of infiltrating immune cells and stromal cells but reduced numbers of tumor cells in the TME compared to patients with lower immune activity. This observation suggests that immune activity is a considerable variable in the liver cancer microenvironment, and it may correlate with MAPK10 expression. This *in silico* finding is potentially important because previous reports demonstrated that immune activity of the TME of HCC patients could affect the efficacy of clinical immunotherapy through the cytotoxic effect and antitumor effect-mediated by TILs (27, 28). Moreover, we evaluated correlation between the immune activity and the expression signatures of specific immune cells in liver cancer microenvironment by CIBERSORTx software (https://cibersortx.stanford.edu/). In line with immune cells of the TME being TILs, macrophages, dendritic cells, and NK cells, the transcriptomic immune activity of the TME uncovered by this study demonstrated significant enrichment of expression signatures representative of CD8^+^ T cells, activated memory CD4^+^ T cells, NK cells, and resting dendritic cells, and the conversion of macrophages from M0 type to M1 type (Wilcoxon test, *P* < 0.05; **Figure 3E**). Additionally, when we ranked liver cancer patients by MAPK10 expression in a descending order, we discovered that HCC patients with high MAPK10 expression had higher immune gene expression, higher scores of tumor infiltrating immune cells and stromal cells, but reduced transcriptomic signatures of hepatocellular carcinoma cancer cells compared to patients with low MAPK10 expression (**Figure 3F**). Consistently, statistical analysis confirmed that HCC patients with medium immune activity had significantly higher expression of MAPK10 than those with low immune activity (Wilcoxon Test, *P* < 0.01; **Figure 3G**), while HCC patients with medium immune activity had a significantly lower expression of MAPK10 compared to patients with high immune activity (Wilcoxon Test, *P* < 0.001; **Figure 3G**). Collectively, these results illustrated that transcriptomically assigned immune activity of liver cancer microenvironment correlates with MAPK10 expression and might potentially be regulated by the MAPK10 kinase. It needs to be noted that further functional tests should be conducted to strengthen out hypothesis of the connection between MAPK10 and the immune activity in the TME of HCC.

**Table 1:** Statistical analysis for the association of MAPK10 expression and the immune activity.

Immune Activity	Expression of MAPK10		Total Number of patients	P value
	Low	High		
Low Activity	152	33	185	<0.001 (Chi square)
Medium Activity	67	41	108	
High Activity	47	34	81	
Total Number	266	108	374	

### Potential Differentially Expressed Immune-Associated Genes Decline in Concomitant With the Decreasing MAPK10 Expression in HCC

**Figure 4:**
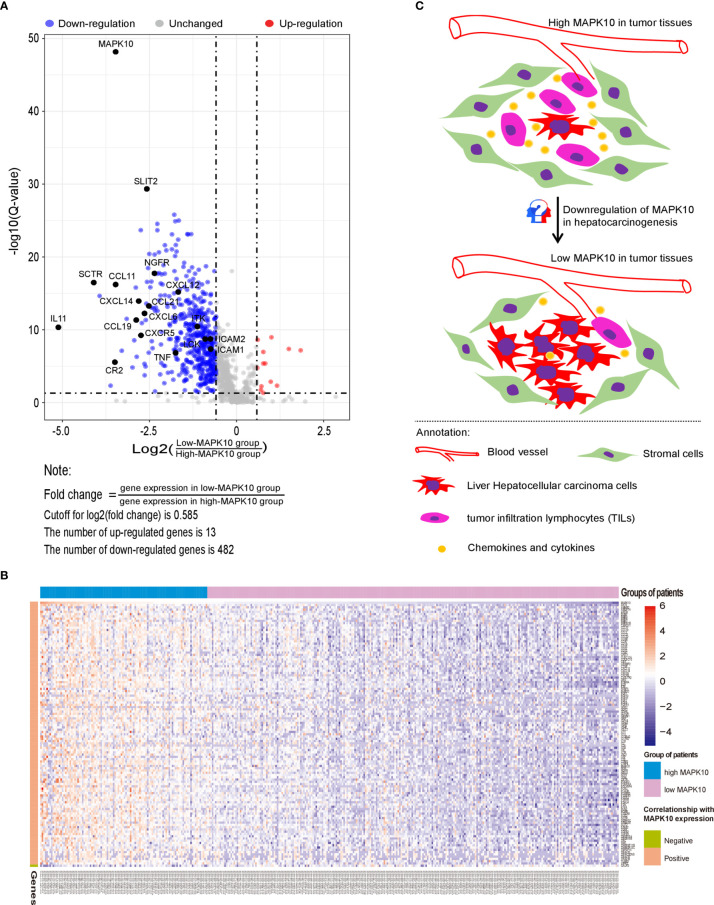
MAPK10 expression correlates with levels of specific immune genes and likely influences the cell composition of the TME in HCC. A gene list containing 2,498 human immune-related genes was obtained from the immunology database and analysis portal (ImmPort) (https://www.immport.org/shared/genelists) (29). As described in the *Materials and Methods* section, 495 differentially expressed immune-associated genes (DIGs) were identified by using cutoff for Q value = 0.05 and cutoff for log_2_ (fold change) = 0.585 **(A)**. Top 117 DIGs were most significantly changed in connection with MAPK10 expression, among which 116 DIGs were positively correlated with MAPK10 expression while one DIG was negatively correlated with MAPK10 expression **(B)** (Cutoff for FDR = 0.05. Cutoff for log_2_ (fold change) = 1.58). Graphical summary assembled in Adobe illustrator demonstrates the composition of the TME of HCC relative to MAPK10 expression: higher numbers of tumor cells and lower numbers of immune cells are expected to be present in MAPK10 low tumors on the basis of the transcriptomic data analysis **(C)**.

Because our previous data indicated that MAPK10 expression might modulate the immune activity in the tumor microenvironment though the recruitment of TILs or TAMs into the TME, we attempted to understand the precise molecular mechanism of this proposed process. For that, a gene list containing 2,498 human immune-related genes was obtained from the immunology database and analysis portal (ImmPort) (https://www.immport.org/shared/genelists) (29). The expression fold changes of individual immune genes were calculated by dividing the mean expression value of a given gene in the low-MAPK10 expression group of HCC patients by the mean expression value in the high-MAPK10 expression group (**Figure 4A**). We next used Q value below 0.05 and average log_2_ (fold change) of at least 0.585 to define differentially expressed genes (DIGs). In this way we found 482 protein coding genes to be significantly down-regulated and 13 genes up-regulated, in concomitant with the decrease of MAPK10 expression, suggesting that these DIGs could be potential targets of MAPK10 (**Figure 4A** and **Supplementary Table S1**). Furthermore, we used a higher cutoff value [log_2_ (fold change) of at least 1.585] to identify 117 most strongly regulated genes (DIGs) in connection to MAPK10 expression (**Supplementary Table 2**). In order to obtain gene expression values of the 117 DIGs in a form of normal distribution and to prepare for the subsequent heat map plotting, we firstly transformed the original expressional values of the 117 DIGs into log2 scale and then transformed them into standardized normal distribution (**Supplementary Table 3**). Using such converted data, we did heat map plotting and found that these 117 DIGs were significantly associated with MAPK10 expression, with 116 DIGs positively and one negatively correlated with the MAPK10 expression (**Figure 4B**). Interestingly, many of the genes expressed in a MAPK10-associated manner were chemokines and cytokines (**Figures 4A, B** and **Table S3**), demonstrating that HCC patients with high MAPK10 expression had increased chemokine and cytokine expression in the TME compared to patients with low MAPK10 expression. In combination with our *in silico* finding that HCC patients with high MAPK10 expression had significantly higher expression scores of stromal cells (**Figures 2A, B**) and infiltrating immune cells (**Figures 2C, D**) but lower scores of tumor cells (**Figures 2E, F**) in their tumor microenvironment compared to patients with low MAPK10 expression, we propose that high expression of MAPK10 in liver cancer tissues might promote the infiltration of immunocytes and the secretion of chemokine and cytokines. High MAPK10 expression may thus act in an anti-tumor manner by promoting immune surveillance in the TME, whereas reduced MAPK10 expression in HCC might facilitate tumor growth due to reduced immune milieu in the TME promoting the evasion of immune surveillance by cancer cells (**Figure 4C**), as indicated by our transcriptome analysis.

### The Biological Function of DIGs Is Consistent With Chemotaxis, Migration, Differentiation and Activation of Leukocytes *via* Intercellular and Intracellular Signaling Pathways

As we identified 495 DIGs being correlated with MAPK10 expression in liver cancer patients, we attempted to elucidate the key biological functions of these DIGs in the TME of HCC patients. Biological function enrichment using Gene Ontology (GO) analysis and KEGG pathway enrichment analysis illustrated that these genes are implicated in cell chemotaxis, leukocyte migration, and positive regulation of response to external stimulus *via* several intercellular or intracellular signaling pathways such as cytokine–cytokine receptor interaction, T cell receptor signaling pathway, and MAPK signaling pathway (**Supplementary Figures 2A, B**). In addition, KEGG pathway enrichment analysis indicated components of tumor necrosis factor (TNF) signaling to be enriched among MAPK10-associated DIGs (**Supplementary Figure 3A**). TNF-α (30–32), TNFR1 (33) and TNFR 2 (34) are well known activators of MAP kinases and as such they could potentially engage MAPK10, promoting the synthesis and secretion of a variety of immune-related mediators such as cytokines IL-6 and IL15, chemokine CXCL15 and CCL2, inflammatory factor PTGS2 and membrane receptor ICAM1 (**Supplementary Figure 3A**). Besides, DIGs could maintain the equilibrium of cellular immunity and humoral immunity through the regulation of Th1 and Th2 differentiation, which was also highlighted by the KEGG analysis of DIGs (**Supplementary Figure S3B**).

### Network Analysis of Protein–Protein Interactions to Search for the MAPK10-Associated Hub Genes in the TME of HCC

As the next step, we researched the potential of specific DIGs to act as the hub genes in the tumor microenvironment of HCC patients.

**Figure 5:**
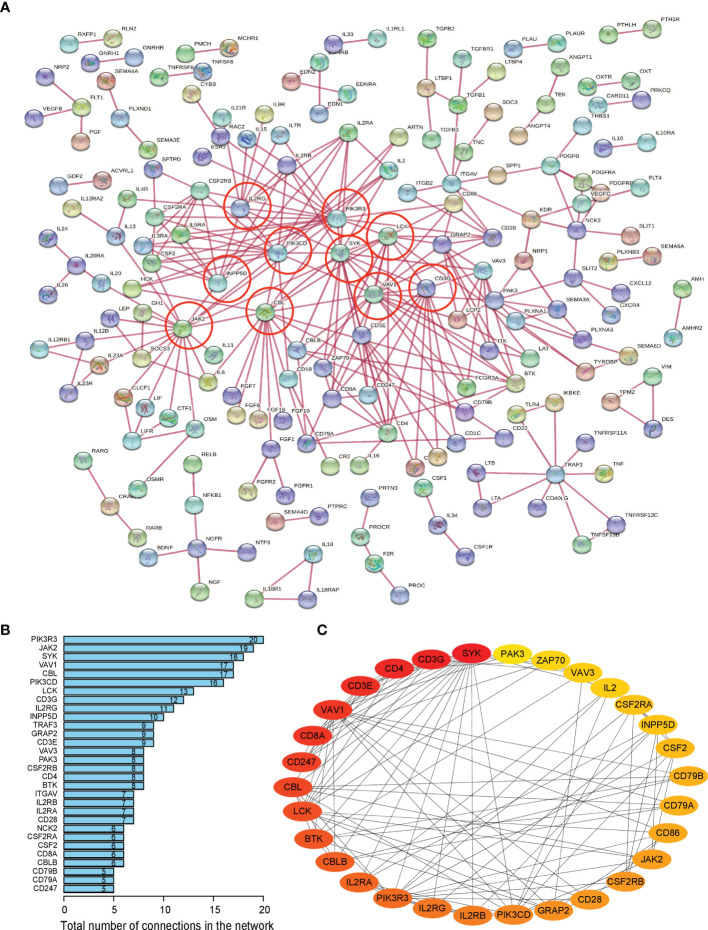
Network analysis of protein–protein interactions (PPIs) revealed hub genes, which putatively mediate the immune effects of MAPK10 in HCC. STRING database was utilized to predict the network of protein–protein interactions (PPIs) for the 495 differentially expressed immune-associated genes **(A)**, and top 10 most connected DIGs were highlighted with red bold circles. The number of connections of each DIG was counted by using the custom R script, and the top 30 most connected DIGs were depicted by bar plotting **(B)**. The algorithm of Maximal Clique Centrality (MCC) in Cytohubba plugin of Cytoscape software was used as an additional method to determine the hub genes among the above identified DIGs **(C)**.

Firstly, protein–protein interactions (PPIs) for the 495 differentially expressed immune-associated genes were predicted by the STRING database (**Figure 5A**). Our search for the top genes, which had the most number of connections in the PPI network returned PIK3R3, JAK2, SYK, VAV1, CBL, PIK3CD, LCK, CD3G, IL2RG, and INPP5D as the most connected hub genes predicted by the STRING database (**Figure 5B**). These top 10 most connected hubs are highlighted with red bold circles in **Figure 5A**. We next used the Cytoscape platform and its dedicated plugins to verify the hubs among 495 DIGs by an additional independent method. Cytoscape is an open source software, which facilitates complex network analysis and provides a variety of plugins to uncover molecular interactions in biological pathways. We utilized the results of the protein–protein interaction (PPI) signaling network predicted by the STRING database (**Figure 5A**) as an input into Cytoscape, followed by processing through the Cytohubba plugin of Cytoscape to search for the hubs in the PPI network. By following the algorithm of Maximal Clique Centrality (MCC) in Cytohubba plugin, we simplified the complex PPI interaction network of 495 DIGs and narrowed down top thirty hub genes (**Figure 5C**). Particularly, the top ten hub genes among them were SYK, CD3G, CD4, CD3E, VAV1, CD8A, CD247, CBL, LCK, and BTK, which showed a clear overlap between STRING and Cytoscape predications (**Figure 5C**). Specifically SYK, CBL, VAV1, LCK, and CD3G genes were identified as hubs by both methods. The analytic score for these top thirty hub genes was obtained through ranking by the MCC method in the Cytohubba plugin of Cytoscape (**Table 2**). Importantly, we found that expression of top thirty hub genes predicted by Cytohubba was positively and significantly correlated with the expression of MAPK10 in HCC patients (Pearson correlation coefficient R^2^ ≥ 0.20, *P* < 0.0001; **Table 3**), suggesting that they were expressed in a MAPK10-associated manner. Collectively, we identified five putative MAPK10-associated hub genes in the liver cancer microenvironment.

**Table 2:** Top 30 hub genes ranked by Maximal Clique Centrality (MCC) method in cytoscape.

Rank	Genes	Score
1	SYK	268
2	CD3G	162
3	CD4	157
4	CD3E	150
5	VAV1	126
6	CD8A	126
7	CD247	120
8	CBL	109
9	LCK	85
10	BTK	78
11	CBLB	72
12	IL2RA	68
13	PIK3R3	67
14	IL2RG	61
15	IL2RB	56
16	PIK3CD	55
17	GRAP2	40
18	CD28	32
19	CSF2RB	32
20	JAK2	31
21	CD86	30
22	CD79A	25
23	CD79B	25
24	CSF2	24
25	INPPP5D	20
26	CSF2RA	20
27	IL2	14
28	VAV3	14
29	ZAP70	12
30	PAK3	11

**Table 3:** Top 30 potential hub genes ranked by Pearson correlation coefficient with MAPK10.

Rank	Genes	R^2^	P value
1	CBL	0.501	4.09 × 10^−25^
2	JAK2	0.471	4.28 × 10^−22^
3	CSF2RB	0.647	1.02 × 10^−21^
4	INPP5D	0.646	2.10 × 10^−21^
5	CBLB	0.457	1.07 × 10^−20^
6	CD4	0.424	9.81 × 10^−18^
7	SYK	0.413	7.17 × 10^−17^
8	VAV3	0.408	2.05 × 10^−16^
9	PIK3R3	0.403	4.76 × 10^−16^
10	CSF2RA	0.397	1.53 × 10^−15^
11	PIK3CD	0.387	7.97 × 10^−15^
12	IL2RB	0.382	1.99 × 10^−14^
13	BTK	0.381	2.37 × 10^−14^
14	CD86	0.376	5.60 × 10^−14^
15	CD3G	0.357	1.16 × 10^−12^
16	VAV1	0.348	4.20 × 10^−12^
17	IL2RA	0.329	7.17 × 10^−11^
18	LCK	0.325	1.20 × 10^−10^
19	CD3E	0.317	3.76 × 10^−10^
20	CD247	0.314	5.07 × 10^−10^
21	GRAP2	0.311	7.99 × 10^−10^
22	IL2RG	0.310	9.32 × 10^−10^
23	IL2	0.301	2.72 × 10^−09^
24	PAK3	0.294	6.76 × 10^−09^
25	CD8A	0.277	4.95 × 10^−08^
26	ZAP70	0.269	1.32 × 10^−07^
27	CD79A	0.262	2.78 × 10^−07^
28	CD79B	0.231	6.26 × 10^−06^
29	CD28	0.208	4.97 × 10^−05^
30	CSF2	0.203	7.90 × 10^−05^

### Identification of the MAPK10-ICAM1 Signaling Axis in the TME of HCC

**Figure 6:**
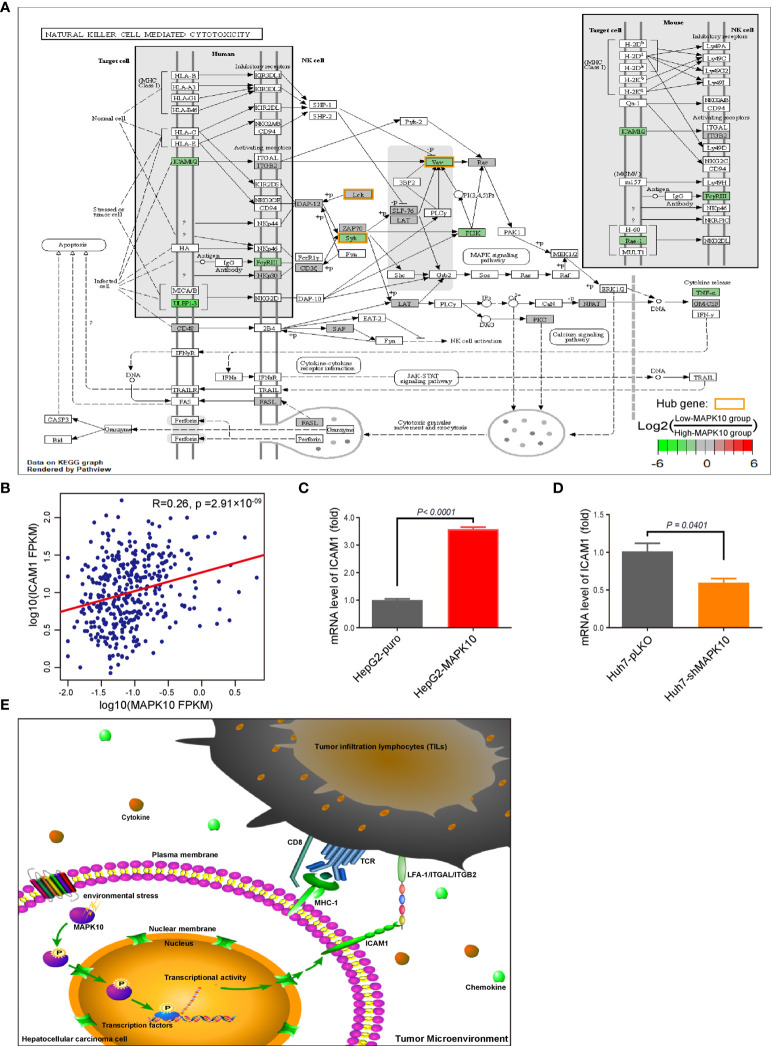
ICAM1 is a putative mediator of MAPK10 effects on immune activity in HCC. As described in the *Materials and Methods* section, our custom R script was used to measure the fold change of immunity-related genes to identify DIGs with average log2(Fold Change) of at least 0.585 and Q value (also known as adjusted *P* value) of less than 0.05. Subsequently, the R package of Pathview was used to visualize the KEGG pathway enrichment analysis of these MAPK10-linked DIGs, highlighting ICAM1 as a potential downstream effector of MAPK10 in the TME. The log2 fold changes of included DIGs are color-coded with green, gray, and red colors; the red-colored genes are up-regulated in low-MAPK10 patients compared to high-MAPK10 patients, while green-colored DIGs are down-regulated. **(A)** The Pearson correlation method was used to correlate MAPK10 expression with ICAM1 expression in HCC patients **(B)** (R^2^ = 0.26, *P* = 2.91 × 10^−9^). **(C)** The mRNA level of ICAM1 was compared between MAPK10-overexpressing HepG2-MAPK10 cell line and its corresponding control HepG2-puro cell line by use of the real time qPCR (Welch’s t test, *P* < 0.0001). **(D)** The mRNA level of ICAM1 was compared between MAPK10-deficient Huh7-shMAPK10 cell line and its corresponding control Huh7-pLKO cell line by use of the real time qPCR (Welch’s t test, *P* = 0.0401). In **(C, D)** one representative result of 3 independent experiments is shown, n=3 in each case. **(E)** Schematic diagram of the key conclusions of this study assembled in Pathway Builder 2.0 Software and Adobe illustrator demonstrates the putative role of MAPK10 in regulating ICAM1 expression in the TME, facilitating the recruitment of immune cells and suppressing cancer immune escape.

By combining the KEGG enrichment analysis of DIGs with the list of hub genes identified through Cytoscape and STRING, we uncovered processes which depend on co-activation of immune cells through Intercellular Adhesion Molecule 1 (ICAM1) to be highlighted (**Figure 6A**). Our pathway analysis using R package of Pathview also showed that, as vital costimulatory molecules in the process of the activation of NK cells, ICAM1 and LFA-1(CD11a/CD18) or ITGAL/ITGB2 could further activate several of the identified hub genes including ZAP70, SYK, VAV1, and LCK in NK cells (**Figure 6A**, hubs in this figure were highlighted with yellow rounded rectangle). This result indicated that ICAM1 might bridge the MAPK10-instigated intercellular signal transduction and the crosstalk between liver cancer and stromal cells and the immunocytes, preventing the evasion of immune surveillance by cancer cells, consistent with previous reports showing that the intracellular interaction between ICAM1 and LFA-1 promotes LFA-1-mediated costimulation in T cells and T cell motility, as well as activation and general adhesion of the leukocytes (9, 35–37). Furthermore, interaction between ICAM1 and LFA-1 is known to activate hereby identified hub genes (ZAP70 and LCK) in T cells (37), suggesting that ICAM1 could act as a core mediator of the proposed capacity of MAPK10 to regulate hub genes in the immunocytes in the TME of HCC patents. Because ICAM1, which plays the key role in the immune cell recruitment and signaling (38, 39), was highlighted by several of our analyses as the potential MAPK10 target gene, we explored the correlation between MAPK10 and ICAM1 expression in HCC patients and found that the expression of MAPK10 was positively correlated with the expression of ICAM1 (R^2^ = 0.26, *P* = 2.91 × 10^−9^; **Figure 6B**). Additionally, we found that high ICAM1 expression indeed correlated with an increase of immune gene expression, and up-regulated expression signatures of immune cells in the TME of HCC patients (**Supplementary Figure 5A**), and with the transcriptomically defined immune activity of the TME **(** **Supplementary Figure 5B** and **Table 4**).

**Table 4:** Statistical analysis for the association of ICAM1 expression and the immune activity in HCC.

Immune Activity	Expression of ICAM1		Total Number of patients	P value
	Low	High		
Low Activity	117	68	185	<0.0001 (Chi square)
Medium Activity	48	60	108	
High Activity	22	59	81	
Total Number	187	187	374	

In order to experimentally verify the regulation of ICAM1 by MAPK10 in the HCC context, we established the lentivirus stably infected MAPK10-overexpressing and deficient HCC cell lines. We used two independent and well characterized HCC cell lines—HepG2 and Huh-7—in this experiment. The mRNA level and protein level of MAPK10 in these cells were detected by real time quantitative PCR and Western blotting respectively. The mRNA level of MAPK10 in lentivirus stably infected HepG2-MAPK10 cells increased significantly when compared with its negative control group of lentivirus stably infected HepG2-puro cells (*P* = 0.0020; **Supplementary Figure 4A**). Subsequently, the protein level of MAPK10 in lentivirus stably infected HepG2-MAPK10 cells increased significantly when compared with its negative control group of lentivirus stably infected HepG2-puro cells (*P* = 0.0401; **Supplementary Figure 4C**). Expression of MAPK10 mRNA in Huh7-shMAPK10 cells was lower than that of Huh7-pLKO control cells (*P* = 0.0941; **Supplementary Figure 4B**), and consistently the protein level of MAPK10 in Huh7-shMAPK10 cells was significantly lower compared to Huh7-pLKO control cells (*P* = 0.0002; **Supplementary Figure 4D**). Our MAPK10 overexpressing and knock-down HCC model lines were thus successfully generated and characterized. We next performed the *in vitro* validation of the ICAM1-MAPK10 connection in these cell lines. We found, by using qPCR, that the expression of ICAM1 in MAPK10-overexpressing HepG2-MAPK10 cell line was significantly higher than in the control HepG2-puro cell line, suggesting that MAPK10 is able to promote the expression of the co-stimulatory molecule ICAM1 in liver cancer cells (*P* < 0.0001; **Figure 6C**). Concomitantly, the expression of ICAM1 in MAPK10-deficient Huh7-shMAPK10 cell line was significantly lower than in the control Huh7-pLKO cell line, suggesting that the silencing of MAPK10 leads to reduced expression of ICAM1 in HCC tumor cell context (*P* = 0.0401; **Figure 6D**). In summary, our results indicated that MAPK10 abundance has a functional impact on the expression of the cell adhesion and costimulatory molecule ICAM1 in liver cancer cells possibly contributing to the MAPK10 immune effects in the TME. We thus hypothesize, on the basis of transcriptomic and *in vitro* data, that the combination between ICAM1 and co-stimulatory molecules such as LFA-1(CD11a/CD18) or ITGAL/ITGB2 could act by activating crucial hub genes in cytotoxic lymphocytes, nature killer cells, TILs and TAMs to promote immune surveillance in the liver cancer microenvironment (**Figure 6E**). Our data demonstrates that MAPK10 expression correlates with ICAM1 expression in the TME of HCC patients, and that MAPK10 is able to functionally influence ICAM1 gene expression in cultured liver cancer cells. We cannot exclude however, that other cell types, which are present in the TME such as stromal cells, also have an impact on the MAPK10-ICAM1 relationship in the TME. Further studies need to be conducted in order to clarify this potentially crucial relationship.

### MAPK10 Levels Have Functional Impact on Stress-Induced Phosphorylation of c-jun at Ser63

We next set upon identifying the potential mechanism, which is utilized by MAPK10 in order to regulate ICAM1 expression. Previous studies found that the transcription factors of the AP-1 family play an important role in activating ICAM1 expression (40–43). Concurrently, AP-1 transcription factors are prominent downstream targets of MAP kinases (44–46). We thus hypothesized that MAPK10 may regulate ICAM1 expression in liver cancer cells by modulating the activity of AP-1TFs. The TF binding site analysis performed *via* two distinct web interfaces—LASAGNA-Search 2.0 database (http://biogrid-lasagna.engr.uconn.edu/lasagna_search/) (47) and PROMO 3.0 database (http://alggen.lsi.upc.es/cgi-bin/promo_v3/promo/promoinit.cgi?dirDB=TF_8.3) (48, 49)—indeed identified binding cites for the c-jun transcription factor in the promoter region of the ICAM1 gene (**Tables 5** and **6**). We next tested if overexpression or knock-down of MAPK10 has an impact on the c-jun activity in the context of HCC. The Ser63 phosphorylation of c-jun was used as a proxy in this analysis, and oxidative stress treatment was used to mimic the highly stressful tumor microenvironment in the *in vitro* setting. We found that c-jun phosphorylation at Ser63 was indeed increased in MAPK10 overexpressing cells (**Supplementary Figures 5C, E**) and it was decreased in MAPK10 knock-down cells (**Supplementary Figures 5D, F**), while c-jun total protein levels remained unaffected in both cases (**Supplementary Figures 5G, H**). Our data thus indicates that MAPK10 may regulate ICAM1 expression *via* c-jun phosphorylation, although further studies need to be conducted in order to functionally strengthen this hypothesis.

**Table 5:** Predicted transcription factor binding sites (TFBSs) of c-jun in the promoter region of the ICAM1 gene in LASAGNA-Search 2.0 database.

Name	Sequence	Position (0-based)	Strand	Score	p-value	E-value
c-jun (T100133)	TCTCCTGCATCAGCCT	441	+	79.31	0.0001	0.239
c-jun (T100133)	TATCTTGGCTCACTGC	398	+	77.46	0.000225	0.54
c-jun (T100133)	AAGCTTGAATCACGGT	1,991	−	76.04	0.0004	0.95

Flanking region that ranges from −2,000 bp upstream to +400 bp downstream of transcription start site (TSS) was selected by default as promoter sequence in LASAGNA-Search 2.0 database. 0-based position means the site of −2,000 bp upstream of TSS.

**Table 6:** Predicted transcription factor binding sites (TFBSs) of c-jun in the promoter region of the ICAM1 gene in PROMO database.

Factor Name	Start Position	End Position	Dissimilarity	String	RE equally	E-value
c-jun [T100133]	392	398	3.244843	TGACTCT	0.29297	0.26635
c-jun [T100133]	771	777	4.129800	TGACTCG	0.29297	0.23312

## Discussion

In this study we analyzed expression data from the pan-cancer TCGA database and found reduced expression of the MAPK10 gene in cancer tissue of HCC patients compared to the surrounding normal tissue. Previous reports indicated that down-regulation of the *MAPK10* gene in the tumor microenvironment promotes the migration and metastasis of breast cancer (50), nasopharyngeal carcinoma (51), and cervical cancer (52). In line with these reports, we found that HCC patients exhibiting lower MAPK10 expression show gene expression profiles consistent with the reduced content of stromal cells and infiltrating immune cells but higher content of tumor cells in the tumor microenvironment compared to patients with higher MAPK10 expression. These features could indicate that reduced MAPK10 expression facilitates cancer progression also in HCC. Consistently, we found that HCC patients with a high level of MAPK10 expression had significantly higher 5-year survival rate than those with a low level of MAPK10 expression. In clinical practice, the most significant complication in late clinical stage patients with liver cancer lies in the metastasis of cancer cells, especially the lung metastasis of liver cancer cells, which migrate *via* blood. Particularly, due to a lack of effective therapy for recurrence and metastasis and immune escape, HCC still remains a devastating malignancy worldwide (2–4). Moreover, it has been reported that the increase of immune activity in the TME induced by immune checkpoint inhibitors targeting PD-1 and CTLA-4 (53, 54), inhibition of immune myeloid-derived suppressor cells (MDSCs) (55), activation of T cells (56), recruitment (57) and infiltration (58, 59) of lymphocytes, polarization of macrophages into M1-like Tumor-associated macrophages (TAMs) (60), stimulation of NK cells and macrophages (61), reduction of regulatory T (Treg) cells population and abrogated suppression of T cells (62) could significantly increase the overall survival and reduce metastasis in different types of cancer. The potential link between MAPK10 and immune activity in the TME of HCC, identified by this study, indicates that targeting MAPK10 in HCC might synergize with the therapeutic efficacy of T cell checkpoint antagonists such as PD-1 antibody.

There are about 20,000 protein coding genes in the human genome, among which there are 2,498 well-known immunity-associated genes that function in various types of immune response (https://www.immport.org/shared/genelists). By analyzing the expression of these 2,498 genes in the TME of HCC patients, we found 495 immune-associated genes to be differentially expressed in concomitant with MAPK10 expression. Out analysis indicated that 482 of these immune-related genes are possibly up-regulated by MAPK10-associated signals while 13 genes are possibly down-regulated. By *in silico* functional analysis of the 495 MAPK10-linked immune-associated DIGs, we found that they are implicated in chemotaxis, migration, differentiation and activation of leukocytes, suggesting that the main function of these differentially expressed genes is likely to recruit immune cells from the blood stream into the TME of HCC. Furthermore, a combination of protein**–**protein interaction (PPI) and hub analysis performed by using STRING and Cytoscape with the KEGG functional enrichment scoring revealed ICAM1 as the putative regulator of MAPK10-associated immune activity in the TME. Consistently, ICAM1 expression declined in concomitant with decreasing MAPK10 expression in HCC patients, and ICAM1 gene expression was functionally linked to MAPK10 levels in cultured HCC cell lines. Moreover, high ICAM1 expression correlated with high immune activity and increased expression of immune genes in the TME of HCC patients. Finally, we identified binding cites for the c-jun transcription factor in the promoter of the ICAM1 gene, and we found that the phosphorylation of c-jun at Ser63 is differentially regulated in MAPK10 overexpressing and knock-down liver cancer cells. This data suggests that MAPK10 may regulate ICAM1 expression by activating c-jun. Importantly, ICAM1 has been reported as a vital co-stimulatory molecule, which contributes to the activation of the hereby identified hub genes (ZAP70 and LCK) in T cells (37). Overall, our analysis in combination with previous data suggests that ICAM1 may be one of key mediators, which bridge MAPK10 levels in the TME with the induction of the hub genes including ZAP70, SYK and LCK and the recruitment and activity of the immunocytes in the TME. Because our findings are based on the *in silico* analysis of transcriptomes of HCC patients in combination with *in vitro* validation in 2 HCC cell lines, further functional studies, such as tumor immune phenotyping and mechanistic validation in additional HCC cell isolates, should be conducted in order to validate these hypotheses. Our study is however instrumental in providing a novel set of potentially important prognostic markers that may aid survival prognosis and therapy selection in the clinical treatment of HCC.

Importantly, it had been reported that expressional levels of ICAM1 serve as a predictor of clinical outcome in several types of cancer including metastatic colorectal cancer (63), indolent non-Hodgkin lymphomas (64), high-grade serous ovarian carcinoma (65) and glioblastoma (66). Within inflamed tissue, ICAM1 was previously found to promote recruitment and survival of immunocytes such as eosinophils and T lymphocytes (67, 68). Moreover, pericytes associated with glioblastoma multiforme (GBM) fatal brain tumors, contribute to the immune-suppressive micro-environment of this cancer by reduced expression of ICAM1 in response to pro-inflammatory cytokine IL-1β (69). The down-regulation of ICAM1 mediated by ectopic expression of miRNA-296-3p in prostate cancer could facilitate metastasis by reducing susceptibility of circulating tumor cells to cytotoxicity by nature killer (NK) cells though inhibition of NK cell activation and expansion (70). These previous observations are consistent with our finding that MAPK10-ICAM1 signaling axis may mediate immune activity and immunocyte numbers also in HCC. Furthermore, adoptive cellular therapy using chimeric antigen receptor (CAR) T cells targeting ICAM1 could effectively eliminate patient-derived anaplastic thyroid cancer cells which were overexpressing ICAM1 on their membrane in animal models (71). In preclinical model, CAR T cells targeting ICAM1 could eliminate advanced thyroid cancer (72). Collectively, ICAM1 has potential to become an import immunotherapeutic target for cancer patients especially *via* adoptive cellular therapy using chimeric antigen receptor (CAR) T cells. Beyond immunotherapeutic treatment in clinical trials in the upcoming future, other patients without malignant tumor could also benefit from immunotherapy targeting ICAM1. As ICAM1 could promote the recruitment and infiltration of leukocytes, blockade of ICAM1 using monoclonal antibodies exerted anti-inflammatory effect in acute pancreatitis, thus attenuating both local pancreatic injury and systemic lung injury (73). Besides, recent study showed that ICAM1 antibody could increase the antibiotic efficacy in the treatment of drug-resistant infections (74).

It has been previously demonstrated that the infiltration of immune cells into the tumor microenvironment at early stages of carcinogenesis can inhibit cancer progression (75–77). However, in the later stages of cancer, the tumor infiltrating immunocytes become tolerant to the tumor environment with higher percentage of suppressive immune cells such as regulatory T cells (T reg) and myeloid-derived suppressor cells (MDSCs) (78–80). Consistently, signals such as MAPK10, which promote the continuous infiltration of immune cells into tumor microenvironment, may improve the prognosis of HCC patients in clinical immunotherapeutic treatment. In a recent clinical trial, cancer patients could not obtain desirable benefit and favorable therapeutic efficacy from immunotherapy using PD-1 antibody and PD-L1 antibody treatment if tumor infiltration lymphocytes were exhausted in the TME (81–84) or if the cancer cells did not express programmed death-ligand 1 (PD-L1) (85–89). Under these or comparable circumstances, boosting of MAPK10-ICAM1 axis in HCC could facilitate immune activity in the TME and support the efficacy of existing immune therapies.

Collectively, we found that MAPK10 expression is reduced in HCC compared to normal liver tissue, and patients with higher MAPK10 expression in HCC have better five-year survival prognosis. We found that MAPK10 expression is functionally linked to the expression of an important co-stimulator and cell adhesion molecule ICAM1 *in vitro*, and the phosphorylation of the c-jun transcription factor known to regulate ICAM1 transcription is also regulated by MAPK10 abundance. We found that increased expression of MAPK10 associates with higher transcriptional scores of immune cells and lower scores of cancer cells in the TME of HCC. Moreover, the expression of multiple immune genes strongly correlated with MAPK10 expression in the tumor microenvironment. We thus propose that MAPK10 and ICAM1 are potential prognostic factors and targets for therapy in HCC, warranting further research of connections between these molecules, immune response and cancer cell survival.

## Data Availability Statement

Publicly available datasets were analyzed in this study. This data can be found here: Original transcriptome sequencing data of a total of 374 HCC sample and their corresponding adjacent normal tissues can be found in The Cancer Genome Atlas (TCGA) database (https://portal.gdc.cancer.gov/). R packages used in this study can be obtained in either Comprehensive R Archive Network (CRAN, https://cran.r-project.org/) or Bioconductor (https://bioconductor.org/). All the datasets generated for this study are included in the article/**Supplementary Material**. The 29 immune marker gene sets used in the ssGSEA analysis to evaluate immune activity can be obtained from the previous publication (20). 

## Author Contributions

HL conceived of the study. LF and HL designed this study. TH and ME gave advices to improve the study. HL made constructions of lentivirus stably infected MAPK10-overexpressing HepG2 cells and MAPK10-deficient Huh7 cells in the Tumor Microenvironment Laboratory of Shenzhen University. HL also performed data analysis using R programming language in homeostasis and stress responses laboratory of Leibniz Institute for Age Research-Fritz Lipmann Institute. YL performed the qPCR experiments to test and verify the conclusion of this study. HL wrote the paper in draft form. LF and ME checked and proofread the paper in final version before submission. All authors contributed to the article and approved the submitted version.

## Funding

This work was supported by the National Natural Science Foundation of China (81772957), the National Key R&D Program of China (2017YFA0503900), the Science and Technology Program of Guangdong Province in China (2019B030301009) and the Industry and Information Technology Foundation of Shenzhen (20180309100135860).

## Conflict of Interest

The authors declare that the research was conducted in the absence of any commercial or financial relationships that could be construed as a potential conflict of interest.

## Publisher’s Note

All claims expressed in this article are solely those of the authors and do not necessarily represent those of their affiliated organizations, or those of the publisher, the editors and the reviewers. Any product that may be evaluated in this article, or claim that may be made by its manufacturer, is not guaranteed or endorsed by the publisher.

## Supplementary Material

Supplementary Figure 1 The schematic diagram for the analysis of MAPK10-associated immune acitvity. R package of GSVA (Gene Set Variation Analysis) using gene-centric single sample Gene Set Enrichment Analysis (ssGSEA) method was utilized to analyze the immune activity of the tumor microenvironment for each cancer patient with HCC. **(A)** Schematic diagram depicts the work flow to evaluate the correlation between immune activity and MAPK10 expression. **(B)** On the basis of immune profiles obtained from GSVA package, HCC patients were classified into three categories through the use of the hierarchical clustering algorithm. **(C)** As described in the *Materials and Methods* section, the three categories (cluster 1, 2 and 3) classified by hierarchical clustering approach were considered to be low immune activity, medium immune activity and high immune activity respectively.

Supplementary Figure 2 Biological function enrichment and pathway enrichment analysis for differentially expressed immune-associated genes using Gene Ontology (GO) analysis and KEGG pathway enrichment analysis. In order to explore the molecular characteristics of the 495 DIGs identified in connection with MAPK10, R package of clusterProfiler was used to perform Gene Ontology (GO) analysis **(A)** and KEGG pathway enrichment analysis **(B)**. The top 10 most significant enrichments of biological functions for the identified DIGs were highlighted by both GO analysis and KEGG pathway enrichment analysis.

Supplementary Figure 3 Visualization of two MAPK10-associated KEGG pathways. As described in the *Materials and Methods* section, our custom R script was used to calculate the fold change of immunity-related genes in low-MAPK10 group when compared to high-MAPK10 group and to identify DIGs with average log2(Fold Change) of at least 0.585 and Q value (also known as adjusted *P* value) of less than 0.05. Subsequently, R package of Pathview was used to visualize the results of the KEGG pathway enrichment analysis for the previously identified DIGs. Two pathways **(A, B)** that involve the highest number of DIGs are presented. The log 2 fold changes of included DIGs are color-coded with green, grey and red colors; the red-colored genes are up-regulated in low-MAPK10 patients compared to high-MAPK10 patients, while green-colored DIGs are down-regulated.

Supplementary Figure 4 Validation of lentivirus stably infected MAPK10-overexpressing HepG2 cells and MAPK10-deficient Huh7 cells. As described in the *Materials and Methods* section, lentivirus stably infected MAPK10-overexpressing HepG2 cells and MAPK10-deficient Huh7 cells were constructed. Then real time qPCR and western blotting were used to test the mRNA and protein level of MAPK10 in these cell lines respectively. **(A)** The mRNA level of MAPK10 was compared between MAPK10-overexpressing HepG2-MAPK10 cell line and its corresponding control HepG2-puro cell line by use of the real time qPCR (Welch’s t test, *P* =0.0020). **(B)** The mRNA level of MAPK10 was compared between MAPK10-deficient Huh7-shMAPK10 cell line and its corresponding control Huh7-pLKO cell line by use of the real time qPCR (Welch’s t test, *P*=0.0941). **(C)** The protein level of MAPK10 was compared between MAPK10-overexpressing HepG2-MAPK10 cell line and its corresponding control HepG2-puro cell line by use of Western Blotting (Welch’s t test, *P* =0.0401). **(D)** The protein level of MAPK10 was compared between MAPK10-deficient Huh7-shMAPK10 cell line and its corresponding control Huh7-pLKO cell line by use of the Western Blotting (Welch’s t test, *P*=0.0002). **(A–D)** show one representative result of 3 independent experiments in each case, n=3 in each experiment.

Supplementary Figure 5 ICAM1 expression is positively correlated with high immune activity and might be regulated by MAPK10-induced phosphorylation of c-jun (Ser63). As described in the *Materials and Methods*, HCC patients were classified into three categories that were corresponding to low, medium, and high immune activity. In order to analyze the link between ICAM1 expression and immune activity, HCC patients were ranked by ICAM1 expression in a descending order **(A)**, and Wilcoxon test was used to correlate immune activity of HCC patients with the expression of ICAM1 (**B** Wilcoxon Test, *P* < 0.05). MAPK10-overexpressing/deficient HCC cell lines were treated with 30 µM of hydrogen peroxide for 4 h. The total protein level of transcription factor c-jun and phosphorylation level of p-c-jun (Ser63) in these cells were detected by Western blotting. **(C)** The protein level of transcription factor c-jun and phosphorylated c-jun (Ser63) were compared between MAPK10-overexpressing HepG2-MAPK10 cell line and its corresponding control HepG2-puro cell line by use of Western blotting. **(D)** The protein level of transcription factor c-jun and phosphorylated c-jun (Ser63) were compared between MAPK10-deficient Huh7-shMAPK10 cell line and its corresponding control Huh7-pLKO cell line by use of the Western blotting. **(E–H)** Quantification of phosphorylated c-jun (Ser63) and total c-jun in **(C, D)** were performed in the software of image J. **(E)** The level of phosphorylated c-jun (Ser63) was compared between MAPK10-overexpressing HepG2-MAPK10 cell line and its corresponding control HepG2-puro cell line (Welch’s t test, *P* < 0.0001). **(F)** The level of phosphorylated c-jun (Ser63) was compared between MAPK10-deficient Huh7-shMAPK10 cell line and its corresponding control Huh7-pLKO cell line (Welch’s t test, *P* = 0.0009). **(G)** The total protein level of c-jun was compared between MAPK10-overexpressing HepG2-MAPK10 cell line and its corresponding control HepG2-puro cell line (Welch’s t test, *P* > 0.05). **(H)** The total protein level of c-jun was compared between MAPK10-deficient Huh7-shMAPK10 cell line and its corresponding control Huh7-pLKO cell line (Welch’s t test, *P* > 0.05). **(C, D)** show one representative result of three independent experiments in each case, n = 3 in each experiment. **P*<0.05; ****P*<0.001; ns, not significant.

Supplementary Table 1 Shows the original expression levels of 495 DIGs in a form of FPKM.

Supplementary Table 2 Shows the original expression level of the most significant 117 DIGs in a form of FPKM.

Supplementary Table 3 Shows the normalized expression level of the most significant 117 DIGs in a form of normal distribution.

## Abbreviations

HCC, Hepatocellular carcinoma cells; MAPK10, Mitogen-Activated Protein Kinase 10; TME, tumor microenvironment; TCGA, The Cancer Genome Atlas database; TCGA-LIHC, TCGA- Liver hepatocellular carcinoma project; ROS, reactive oxygen species; TILs, tumor infiltration lymphocytes; CAFs, Carcinoma-Associated Fibroblasts; FPKM, fragments per kilobase of exon model per million reads mapped; DIGs, differentially expressed immune-associated genes; ICAM1, Intercellular Adhesion Molecule-1; GO, Gene oncology; KEGG, Kyoto Encyclopedia of Genes and Genomes; GSVA, Gene Set Variation Analysis; ssGSEA, single sample gene set enrichment analysis; CAR, chimeric antigen receptor; PPI, Protein-Protein Interaction; TMB, Tumor Mutation Burden; TAMs, tumor-associated macrophages; TNF, tumor necrosis factor; OCT3, organic cation transporter 3; ImmPort, immunology database and analysis portal; STRING, Search Tool for the Retrieval of Interacting Genes/Proteins; RT-PCR, reverse transcription-polymerase chain reaction; qPCR, quantitative real-time polymerase chain reaction; MCC, Maximal Clique Centrality; CTCs, circulating tumor cells; GM-CSF, Granulocyte-Macrophage Colony-Stimulating Factor; GBM, Glioblastoma multiforme; MSRS, Maximally Selected Rank Statistics; HR, hazard ratio; MDSCs, myeloid-derived suppressor cells; TIMER 2.0, Tumor IMmune Estimation Resource 2.0; CTLA-4, cytotoxic T lymphocyte-associated protein 4; PD-1, programmed cell death protein 1; PD-L1, programmed cell death-ligand 1; TFBSs, Predicted Transcription factor binding sites; TSS, transcription start site.
