# Supplementary figures and images for: MAPK10 Expression as a Prognostic Marker of the Immunosuppressive Tumor Microenvironment in Human Hepatocellular Carcinoma
Source: Front Oncol. 2021 Aug 2;11:687371. doi: 10.3389/fonc.2021.687371 (PMC8366563; doi:10.3389/fonc.2021.687371)

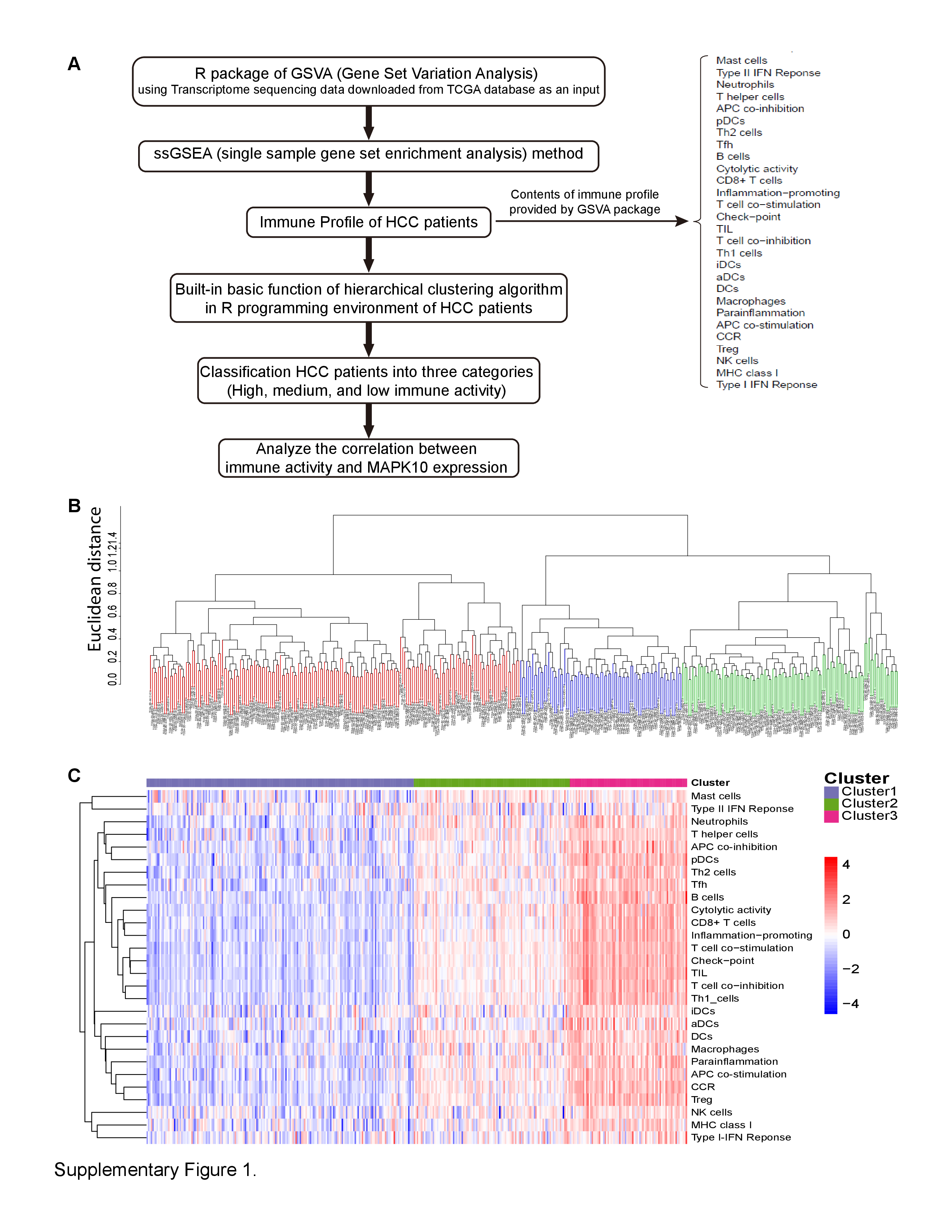

Supplement: Supplementary Figure 1 — The schematic diagram for the analysis of MAPK10-associated immune acitvity. R package of GSVA (Gene Set Variation Analysis) using gene-centric single sample Gene Set Enrichment Analysis (ssGSEA) method was utilized to analyze the immune activity of the tumor microenvironment for each cancer patient with HCC. (A) Schematic diagram depicts the work flow to evaluate the correlation between immune activity and MAPK10 expression. (B) On the basis of immune profiles obtained from GSVA package, HCC patients were classified into three categories through the use of the hierarchical clustering algorithm. (C) As described in the Materials and Methods section, the three categories (cluster 1, 2 and 3) classified by hierarchical clustering approach were considered to be low immune activity, medium immune activity and high immune activity respectively. [file DataSheet_1.zip › SupplementaryMaterial/Supplementary Figure 1.tiff]

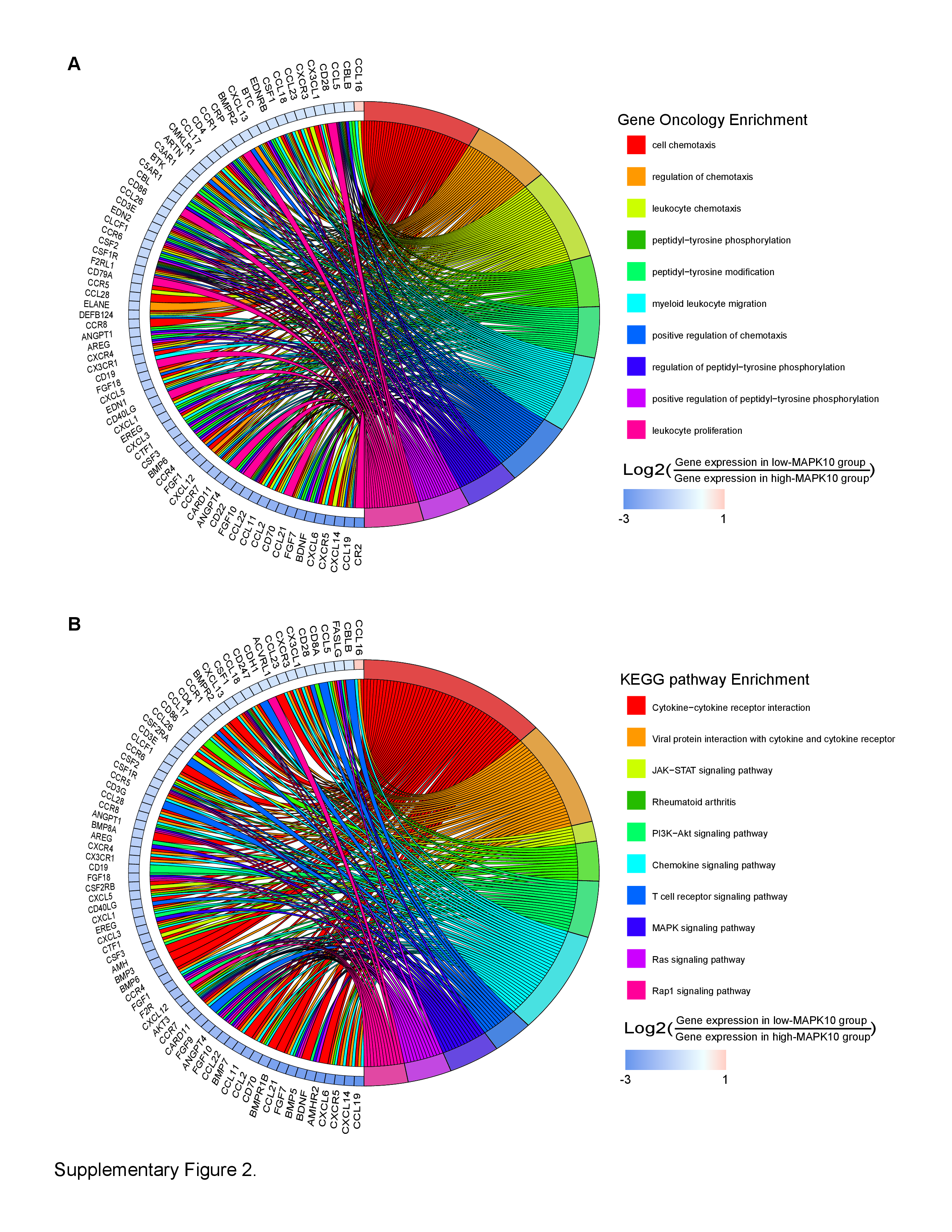

Supplement: Supplementary Figure 1 — The schematic diagram for the analysis of MAPK10-associated immune acitvity. R package of GSVA (Gene Set Variation Analysis) using gene-centric single sample Gene Set Enrichment Analysis (ssGSEA) method was utilized to analyze the immune activity of the tumor microenvironment for each cancer patient with HCC. (A) Schematic diagram depicts the work flow to evaluate the correlation between immune activity and MAPK10 expression. (B) On the basis of immune profiles obtained from GSVA package, HCC patients were classified into three categories through the use of the hierarchical clustering algorithm. (C) As described in the Materials and Methods section, the three categories (cluster 1, 2 and 3) classified by hierarchical clustering approach were considered to be low immune activity, medium immune activity and high immune activity respectively. [file DataSheet_1.zip › SupplementaryMaterial/Supplementary Figure 2.tiff]

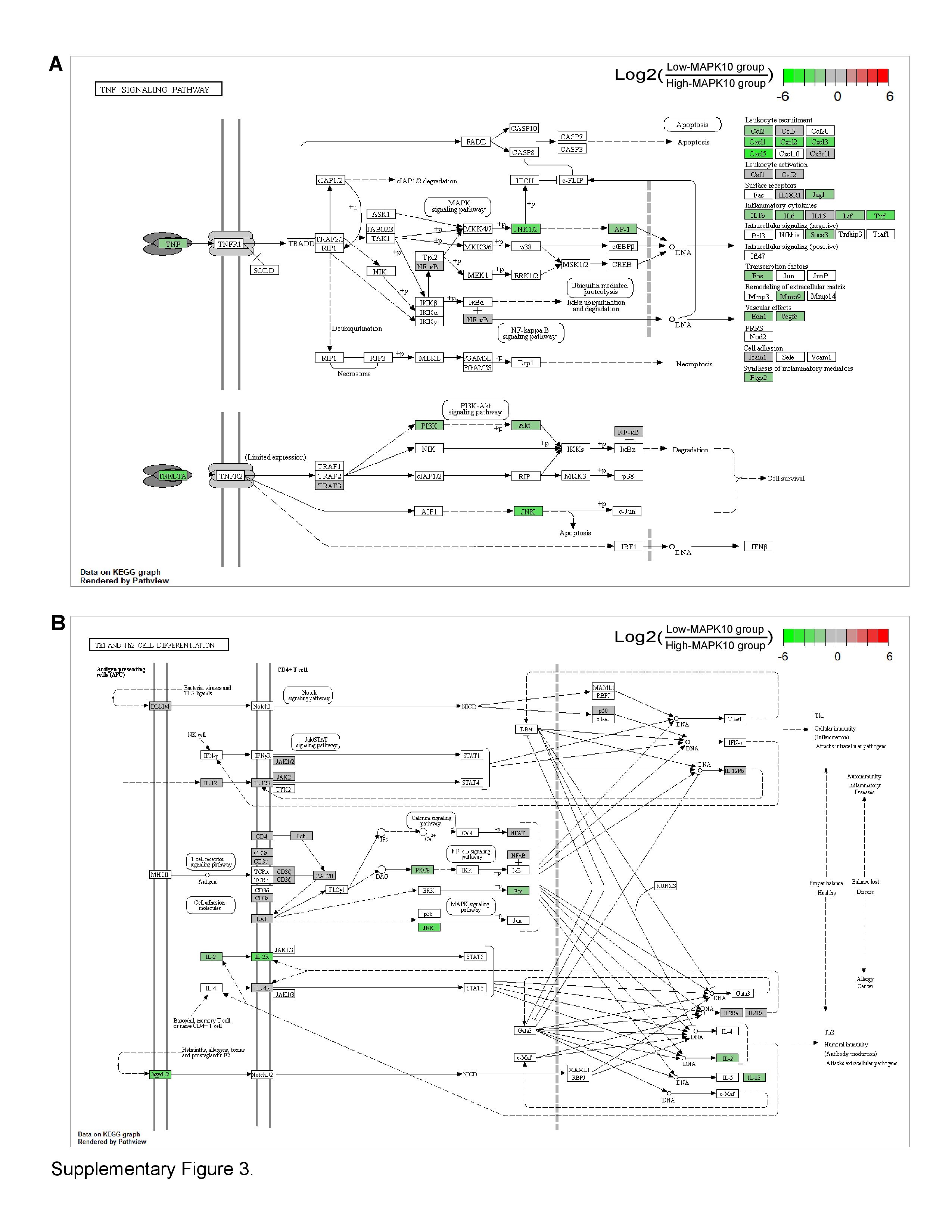

Supplement: Supplementary Figure 1 — The schematic diagram for the analysis of MAPK10-associated immune acitvity. R package of GSVA (Gene Set Variation Analysis) using gene-centric single sample Gene Set Enrichment Analysis (ssGSEA) method was utilized to analyze the immune activity of the tumor microenvironment for each cancer patient with HCC. (A) Schematic diagram depicts the work flow to evaluate the correlation between immune activity and MAPK10 expression. (B) On the basis of immune profiles obtained from GSVA package, HCC patients were classified into three categories through the use of the hierarchical clustering algorithm. (C) As described in the Materials and Methods section, the three categories (cluster 1, 2 and 3) classified by hierarchical clustering approach were considered to be low immune activity, medium immune activity and high immune activity respectively. [file DataSheet_1.zip › SupplementaryMaterial/Supplementary Figure 3.tiff]

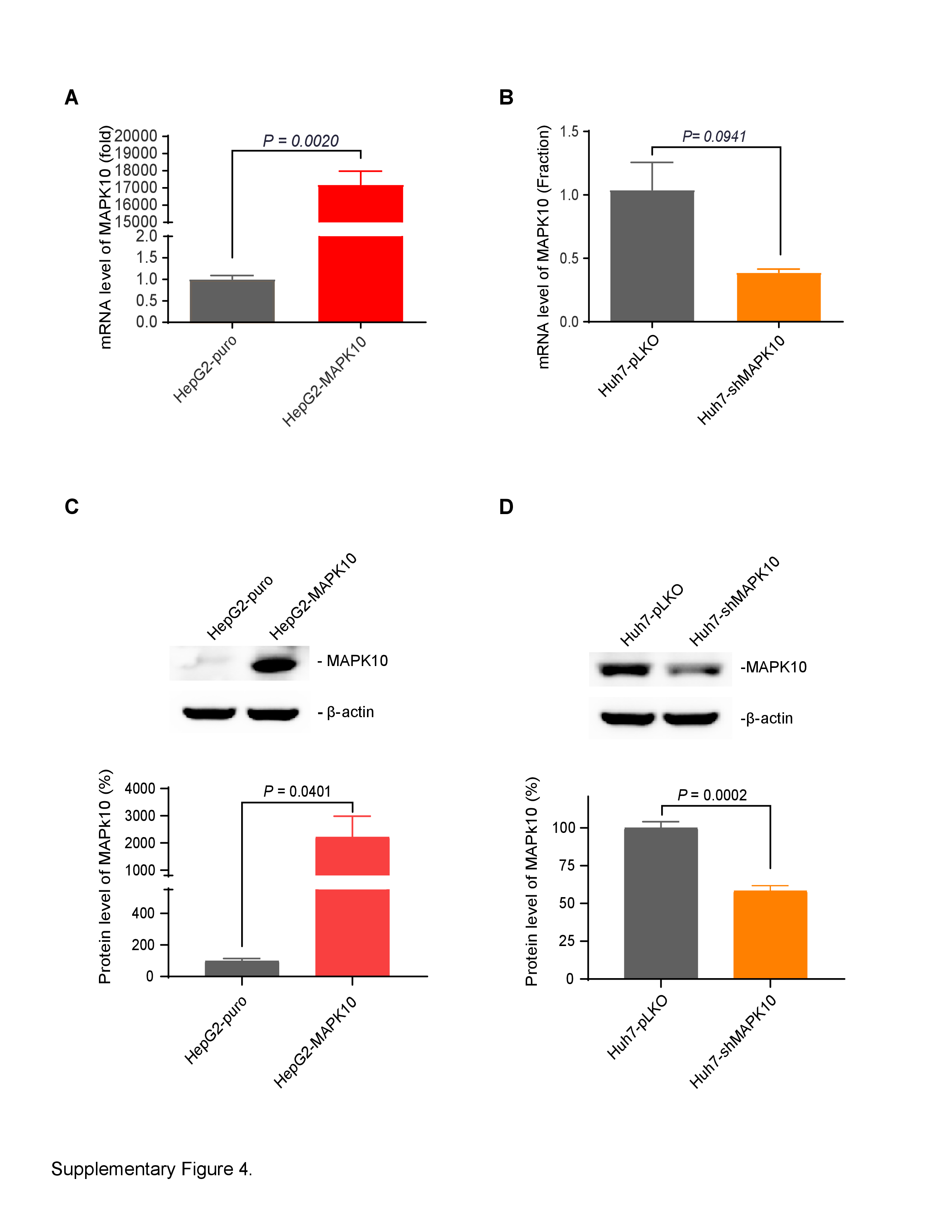

Supplement: Supplementary Figure 1 — The schematic diagram for the analysis of MAPK10-associated immune acitvity. R package of GSVA (Gene Set Variation Analysis) using gene-centric single sample Gene Set Enrichment Analysis (ssGSEA) method was utilized to analyze the immune activity of the tumor microenvironment for each cancer patient with HCC. (A) Schematic diagram depicts the work flow to evaluate the correlation between immune activity and MAPK10 expression. (B) On the basis of immune profiles obtained from GSVA package, HCC patients were classified into three categories through the use of the hierarchical clustering algorithm. (C) As described in the Materials and Methods section, the three categories (cluster 1, 2 and 3) classified by hierarchical clustering approach were considered to be low immune activity, medium immune activity and high immune activity respectively. [file DataSheet_1.zip › SupplementaryMaterial/Supplementary Figure 4.tiff]

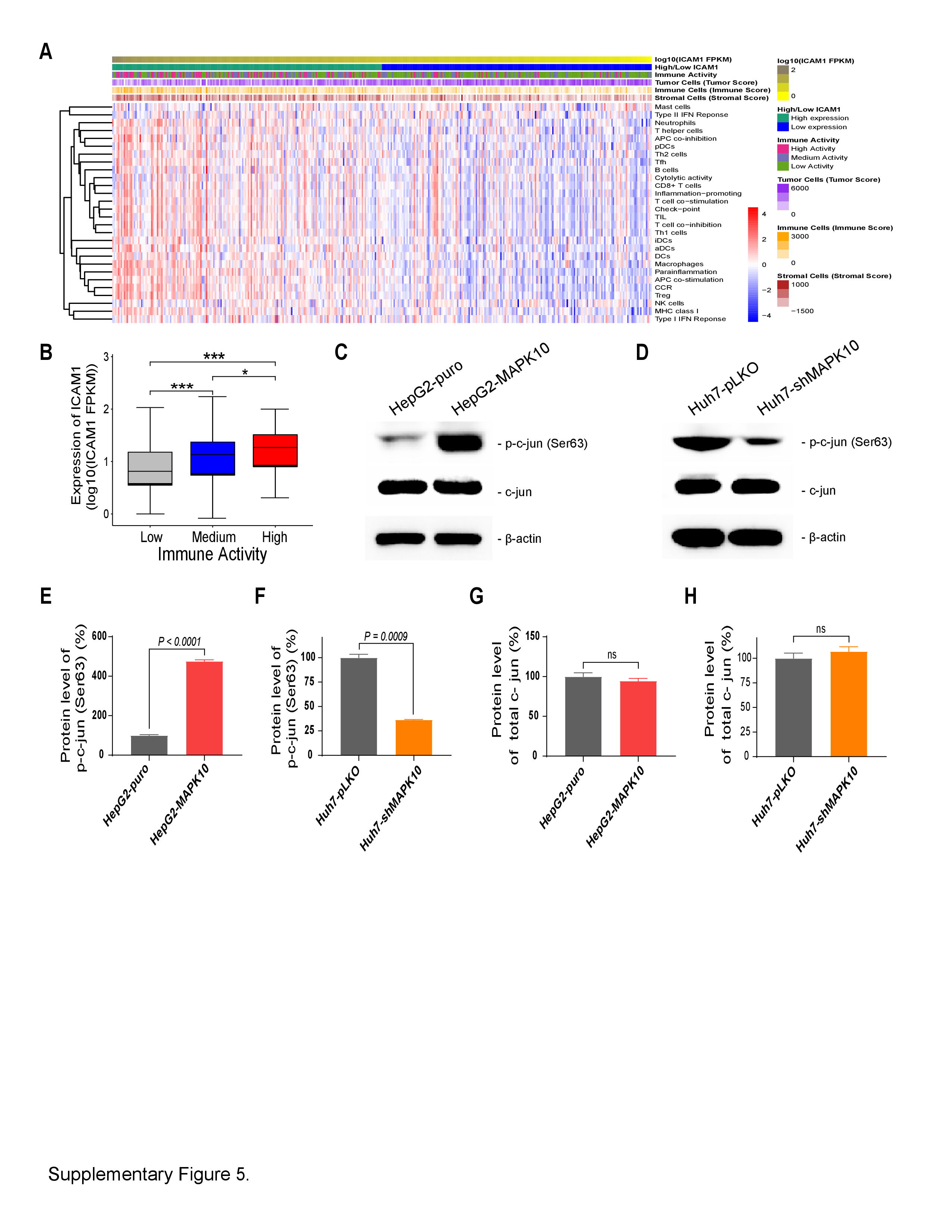

Supplement: Supplementary Figure 1 — The schematic diagram for the analysis of MAPK10-associated immune acitvity. R package of GSVA (Gene Set Variation Analysis) using gene-centric single sample Gene Set Enrichment Analysis (ssGSEA) method was utilized to analyze the immune activity of the tumor microenvironment for each cancer patient with HCC. (A) Schematic diagram depicts the work flow to evaluate the correlation between immune activity and MAPK10 expression. (B) On the basis of immune profiles obtained from GSVA package, HCC patients were classified into three categories through the use of the hierarchical clustering algorithm. (C) As described in the Materials and Methods section, the three categories (cluster 1, 2 and 3) classified by hierarchical clustering approach were considered to be low immune activity, medium immune activity and high immune activity respectively. [file DataSheet_1.zip › SupplementaryMaterial/Supplementary Figure 5.tiff]
